# Metabolic rewiring controlled by HIF-1α tunes IgA-producing B-cell differentiation and intestinal inflammation

**DOI:** 10.1038/s41423-024-01233-y

**Published:** 2024-11-14

**Authors:** Xianyi Meng, Sahar Asadi-Asadabad, Shan Cao, Rui Song, Zhen Lin, Mohammed Safhi, Yi Qin, Estelle Tcheumi Tactoum, Verena Taudte, Arif Ekici, Dirk Mielenz, Stefan Wirtz, Georg Schett, Aline Bozec

**Affiliations:** 1 Department of Internal Medicine 3, Friedrich-Alexander-University Erlangen-Nürnberg (FAU) and Universitätsklinikum Erlangen, Erlangen, 91054 Germany; 2 Deutsches Zentrum Immuntherapie (DZI), Friedrich-Alexander-University Erlangen-Nürnberg (FAU) and Universitätsklinikum Erlangen, Erlangen, 91054 Germany; 3 Institute of Experimental and Clinical Pharmacology and Toxicology, Friedrich-Alexander-Universität Erlangen-Nürnberg, Erlangen, 91054 Germany; 4 Insitute of Laboratory Medicine, Philipps University of Marburg, Marburg, 35043 Germany; 5 Institute of Human Genetics, Friedrich-Alexander-University Erlangen-Nürnberg (FAU) and Universitätsklinikum Erlangen, Erlangen, 91054 Germany; 6 Division of Molecular Immunology, Friedrich-Alexander-University Erlangen-Nürnberg (FAU) and Universitätsklinikum Erlangen, Erlangen, 91054 Germany; 7 Department of Internal Medicine 1, Friedrich-Alexander-University Erlangen-Nürnberg (FAU) and Universitätsklinikum Erlangen, Erlangen, 90154 Germany

**Keywords:** Hypoxia, B cells, IgA, HIF-1α, Intestinal inflammation, Lymphocytes, Mechanisms of disease

## Abstract

Germinal centers where B cells undergo clonal expansion and antibody affinity maturation are hypoxic microenvironments. However, the function of hypoxia-inducible factor (HIF)-1α in immunoglobulin production remains incompletely characterized. Here, we demonstrated that B cells lacking HIF-1α exhibited significantly lower glycolytic metabolism and impaired IgA production. Loss of HIF-1α in B cells affects IgA-producing B-cell differentiation and exacerbates dextran sodium sulfate (DSS)-induced colitis. Conversely, promoting HIF-1α stabilization via a PHD inhibitor roxadustat enhances IgA class switching and alleviates intestinal inflammation. Mechanistically, HIF-1α facilitates IgA class switching through acetyl-coenzyme A (acetyl-CoA) accumulation, which is essential for histone H3K27 acetylation at the Sα region. Consequently, supplementation with acetyl-CoA improved defective IgA production in *Hif1a*-deficient B cells and limited experimental colitis. Collectively, these findings highlight the critical importance of HIF-1α in IgA class switching and the potential for targeting the HIF-1α-dependent metabolic‒epigenetic axis to treat inflammatory bowel diseases and other inflammatory disorders.

## Introduction

Germinal centers (GCs) are essential for the production of long-lasting plasma cells that generate antibodies and memory B cells [1]. In these structures, B cells undergo somatic hypermutation (SHM) and class-switch recombination (CSR) [2]. CSR is a process that merges a downstream constant region (Cγ3, Cγ1, Cγ2b, Cγ2c, Cε or Cα) to the V(D)J region, converting an Ig molecule from IgM to IgG3, IgG1, IgG2b, IgG2c or IgA. It generally links the donor switch region (Sμ) and one of the downstream acceptor switch regions (Sγ3, Sγ1, Sγ2b, Sγ2c, Sε or Sα) in the *Igh* locus [3]. Epigenetic histone modifications provide crucial signals for transcriptional accessibility, germline transcription, R loop formation, AID recruitment and AID-induced DNA double-strand breaks (DSBs) during CSR [4, 5]. Notably, increasing evidence supports a significant connection between epigenetic alterations and cell differentiation metabolism [6].

Although recent studies have reported that GC areas are hypoxic [7, 8], the precise role of hypoxia-inducible factor (HIF)-1α in the antibody response remains unclear. Under normoxic conditions, HIF-1α is continuously hydroxylated by prolyl hydroxylase domain-containing enzymes (PHDs), which in turn recruit the von Hippel‒Lindau protein (VHL) for HIF-1α ubiquitination and degradation [9]. Under hypoxic conditions, PHDs are inhibited, resulting in the stabilization of HIF-1α and the expression of genes involved in metabolism, angiogenesis and erythropoiesis [10]. With respect to B cells, a recent study revealed that IgG1 class switching and CD138 expression were accelerated under hypoxic conditions (1% O_2_) [11]. However, other studies have indicated that GC B-cell formation [12], IgG2c antibody production and memory B-cell differentiation [7] are impaired due to HIF-1α stabilization after the deletion of VHL in B cells. Although the basis for this discrepancy is unclear, it seems that HIF-1α signaling is involved in the regulation of GC B-cell activation and the antibody response.

Immunoglobulin A (IgA), the most abundant mucosal immunoglobulin, regulates the composition and function of the gut microbiota [13, 14]. It primarily has a noninflammatory function that serves mucosal protection and gut homeostasis [15]. Following immunological interactions between dendritic cells (DCs), T cells, and B cells in Peyer’s patches (PPs), activated B cells undergo a class switch to IgA-producing cells in gut-associated lymphoid tissues (GALTs) [16]. Secretory IgA then participates in the crosstalk between the host and its intestinal contents, ensuring an appropriate microbiome and intestinal homeostasis [17]. IgA-deficient mice exhibit persistent intestinal inflammation and are hypersusceptible to dextran sodium sulfate (DSS)-induced colonic damage [18, 19]. Furthermore, exogenous IgA administration mitigates colitis in experimental colitis models, suggesting that IgA might be a therapeutic strategy for intestinal diseases [20–22].

In this study, we observed an unforeseen metabolic function of HIF-1α in IgA-producing B-cell differentiation. Our findings shed light on the crucial metabolic influence of HIF-1α on the IgA response in controlling intestinal inflammation during DSS-induced colitis. PHD inhibitor treatment or acetyl-CoA supplementation upregulated IgA production and protected mice from DSS-induced colitis. These findings highlight the PHD/HIF-1α axis as a prospective target for treating inflammatory bowel diseases.

## Results

### Loss of HIF-1α in B cells leads to impaired IgA-producing cell differentiation

**Fig. 1:**
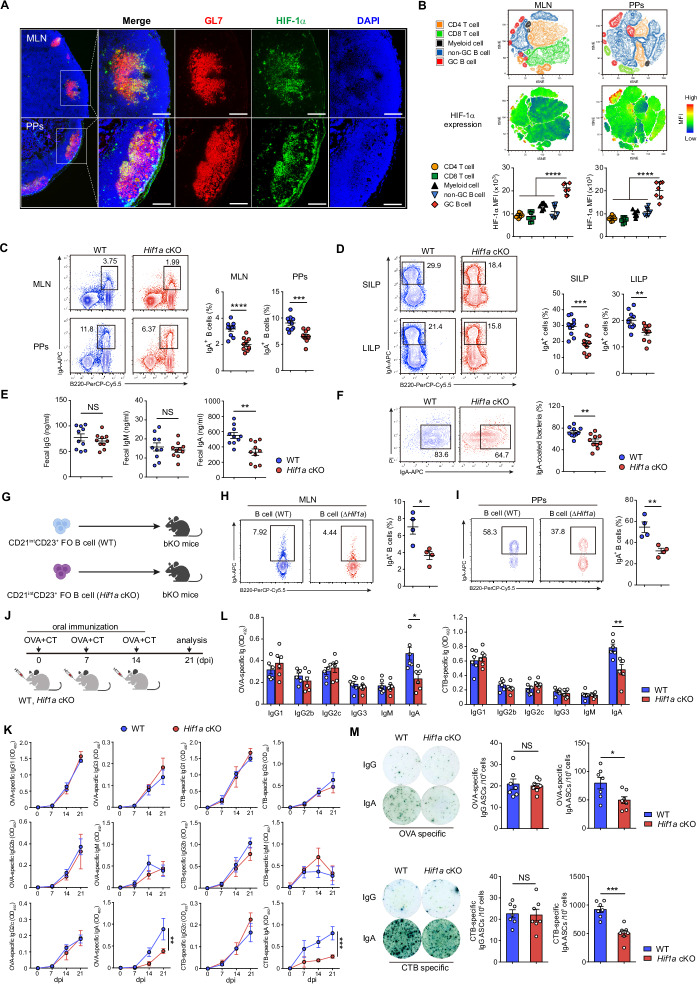
**Loss of HIF-1α in B cells leads to impaired IgA-producing cell differentiation**. **A** Representative immunofluorescence microscopy images of MLN and PP sections from 8-week-old C57BL/6 WT mice (HIF-1α, green; GL7, red; DAPI, blue). Scale bars, 100 μm. **B** Representative tSNE map of clusters identified by FlowSOM and tSNE projection of HIF-1α expression in CD45^+^ lymphocytes from MLNs and PPs of 8-week-old C57BL/6 WT mice (top). Quantification of HIF-1α expression in CD4 T cells (CD45^+^CD4^+^), CD8 T cells (CD45^+^CD8^+^), myeloid cells (CD45^+^CD11b^+^Gr1^+^), non-GC B cells (CD45^+^CD19^+^Fas^-^GL7^-^) and GC B cells (CD45^+^CD19^+^Fas^+^GL7^+^) in MLNs and PPs from 8-week-old C57BL/6 WT mice (*n* = 7) via flow cytometry (bottom). **C** Flow cytometry plots and quantification of B220^+^IgA^+^ B cells in MLNs and PPs from WT and *Hif1a* cKO mice (*n* = 10 per group). **D** Flow cytometry plots and quantification of B220^-^IgA^+^ cells in the small intestine lamina propria (SILP) and large intestine lamina propria (LILP) from WT and *Hif1a* cKO mice (*n* = 10 per group). **E** IgG, IgM and IgA levels in the cecal contents of WT and *Hif1a* cKO mice were measured via ELISA (*n* = 10 per group). **F** Representative plots and frequencies of IgA-coated bacteria in the small intestine of WT and *Hif1a* cKO mice (*n* = 10 per group). **G** Schematic of the adoptive transfer experiment showing that sorted CD21^int^CD23^+^ FO B cells from WT and *Hif1a* cKO mice were injected into bKO mice. **H**, **I** Flow cytometry plots and quantification of IgA^+^ B cells in donor cells from the MLN and PPs of recipient mice (*n* = 4 per group). **J** Experimental schedule for OVA plus CT oral immunization. The mice were orally immunized with OVA plus CT on days 0, 7 and 14 and analyzed on day 21 after the first immunization. dpi, days post immunization. **K** Serum was collected from *Hif1a* cKO and littermate control mice (*n* = 4 per group) on days 0, 7, 14, and 21 postimmunization to detect OVA- or CTB-specific IgG1, IgG2b, IgG2c, IgG3, IgM and IgA via ELISA. **L** Feces were collected from *Hif1a* cKO mice and littermate control mice (*n* = 6 per group) on day 21 after the first immunization to detect OVA- or CTB-specific IgG1, IgG2b, IgG2c, IgG3, IgM and IgA by ELISA. **M** Mononuclear cells were isolated from the iLP of *Hif1a* cKO mice and littermate control mice (*n* = 7 per group) on day 21 after the first immunization to quantify OVA- or CTB-specific IgG-secreting cells and IgA-secreting cells via ELISPOT. The data represent the means ± SEMs. The results are representative of three independent experiments. *p* values were calculated via one-way ANOVA followed by Dunnett’s test (**B**), two-way analysis of variance with Bonferroni post hoc correction for paired data (**K**) or an unpaired two-tailed Student’s *t* test (**C**-**F**, **H**, **I**, **L**, **M**). *p < 0.05, **p < 0.01, ***p < 0.001, ****p < 0.0001. NS not significant (*p* > 0.05)

To elucidate the specific role of HIF-1α signaling in the humoral response, we sought to delineate its expression in the germinal center (GC) of mesenteric lymph nodes (MLNs) and Peyer’s patches (PPs). We used immunofluorescence staining of HIF-1α together with the GC marker GL7. HIF-1α was highly expressed in the GL7^+^ GC area of MLNs and PPs from C57BL/6 WT mice (Fig. 1A; Supplementary Fig. 1A). In addition, flow cytometry profiling of HIF-1α expression in immune cell populations from MLNs and PPs revealed higher HIF-1α expression in GC B cells than in non-GC B cells or other immune cell populations, including CD4 T cells, CD8 T cells and myeloid cells (Fig. 1B; Supplementary Fig. 1B, C). These data suggest that HIF-1α is highly expressed in GC B cells in the steady state.

To understand the effect of HIF-1α stabilization on antibody production in vivo, we used mice deficient in HIF-1α, specifically in B cells (*Hif1a* conditional knockout (cKO) mice). The analyses of IgG, IgM and IgA antibody-secreting cells (ASCs) in the bone marrow (BM), spleen (SP) and MLN by ELISPOT revealed that the loss of HIF-1α in B cells specifically led to fewer IgA-secreting cells (Supplementary Fig. 2A). In addition, reduced IgA antibody levels were also detected in the serum of *Hif1a* cKO mice, whereas the levels of IgG, IgG1, IgG2b, IgG2c, IgE and IgM were unaffected (Supplementary Fig. 2B). IgA is the major Ig isotype in the intestinal mucosa, where it controls bacterial translocation or neutralizes bacterial toxins [23, 24]. Thus, we focused our study on IgA-producing cells in gut-associated lymphoid tissues (GALTs). Few IgA-producing cells were observed in the GALT from *Hif1a* cKO mice (Fig. 1C, D). We next evaluated the Ig levels and IgA-binding bacteria in the cecal contents. Secreted IgA, but not IgG or IgM, was decreased in the stool of *Hif1a* cKO mice (Fig. 1E). Consistent with this observation, IgA-coated bacteria were reduced in *Hif1a* cKO mice (Fig. 1F). These data demonstrate that HIF-1α is crucial for IgA antibody production in vivo.

We next studied whether *Hif1a*-deficient B cells have a defective ability to produce IgA in the physiological environment. To achieve this goal, an adoptive transfer experiment was conducted. CD23^int^CD21^+^ Follicular (FO) B cells were sorted from WT or *Hif1a* cKO mice and transferred into B-cell-deficient hosts (bKO mice). After two weeks of reconstitution, IgA^+^ B cells were assessed in the MLN and PPs of the recipient mice (Fig. 1G). Compared with WT FO B cells, *Hif1a*-deficient FO B cells resulted in fewer IgA^+^ B cells (Fig. 1H, I). Additionally, the results from the cell proliferation and viability analyses demonstrated comparable proliferation and survival rates of WT and *Hif1a*-deficient IgA^+^ B cells in the MLN and PPs (Supplementary Fig. 2C, D).

We have previously shown that *Hif1a* cKO mice display normal antigen-specific IgM, IgG1 and IgG3 levels in T-cell-dependent or T-cell-independent immunization models [25]. However, the production of antigen-specific IgA has not been investigated in *Hif1a* cKO mice after immunization. Therefore, we orally immunized *Hif1a* cKO and littermate control mice with ovalbumin (OVA) plus cholera toxin (CT) (Fig. 1J). Despite similar levels of antigen-specific IgG1, IgG2b, IgG2c, IgG3 or IgM, *Hif1a* cKO mice exhibited reduced production of IgA against OVA and the B subunit of CT (CTB) compared with control mice (Fig. 1K). Accordingly, *Hif1a* cKO mice presented lower levels of OVA- or CTB-specific fecal IgA and fewer OVA- or CTB-specific IgA ASCs in the intestinal lamina propria (iLP) than their littermate control mice (Fig. 1L, M). Together, these data demonstrate the distinctive role of HIF-1α in IgA production.

### HIF-1α-dependent glycolytic metabolism controls IgA production in B cells

To study the regulatory mechanism of IgA antibody production by HIF-1α, the percentages of GC B cells (GL7^+^Fas^+^B220^+^CD19^+^) and plasma cells (CD138^+^B220^-^) in the BM, SP, MLN and PPs from *Hif1a* cKO and littermate control mice were measured via flow cytometry. No significant difference was detected in the number of GC B cells or plasma cells in *Hif1a* cKO mice (Supplementary Fig. 3A, B), indicating that HIF-1α was dispensable for the differentiation of these populations. Given that CCR6 expression and TGF-β signaling in B cells are essential for IgA^+^ B-cell trafficking and differentiation in PPs [26, 27], we analyzed CCR6 and pSmad2/3 levels in IgA^+^ B cells. The expression levels of CCR6 and pSmad2/3 in total IgA^+^ B cells or IgA^+^CCR1^+^GL7^-^ subepithelial dome (SED) B cells from *Hif1a* cKO mice were comparable to those in WT control mice (Supplementary Fig. 3C, D). In addition, the numbers of follicular helper T (Tfh) cells, T follicular regulatory (Tfr) cells, follicular dendritic cells (FDCs) and αVβ8^+^ DCs, which may influence B-cell activation in GCs [28], were unchanged in the MLNs and PPs of *Hif1a* cKO mice (Supplementary Fig. 3E–G). These data suggest that the regulation of IgA-producing B-cell differentiation by the transcription factor HIF-1α is dependent on a cell intrinsic mechanism.

**Fig. 2:**
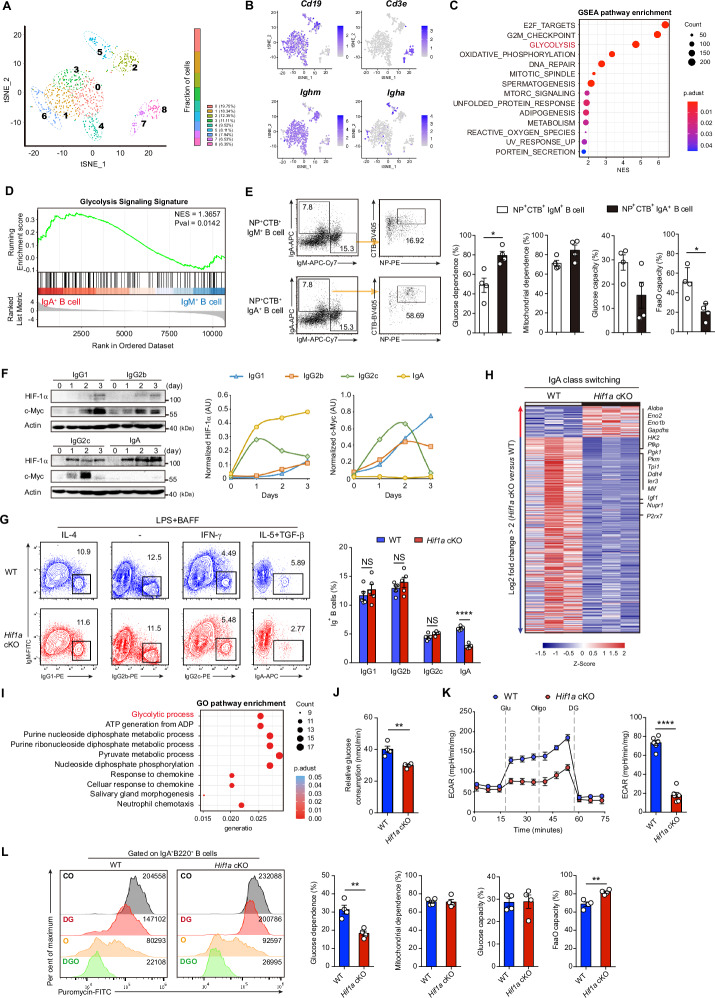
**HIF-1α regulates IgA production in B cells through glycolytic metabolism**. **A** tSNE map of CD45^+^ lymphocytes sorted from PPs of 8-week-old WT mice by cluster (left) and cluster proportions (right). **B** tSNE colored according to the expression of the marker genes *Cd19*, *Cd3e*, *Ighm*, and *Igha* in these identified clusters. **C** GSEA enrichment analysis of the top pathways significantly upregulated in IgA^+^ B-cell clusters (5, 6) compared with IgM^+^ B-cell clusters (0–4). **D** GSEA plot of the glycolysis pathway enriched in IgA^+^ B-cell clusters compared with IgM^+^ B-cell clusters. **E** Flow cytometry plots of NP^+^CTB^+^IgM^+^ B cells and NP^+^CTB^+^IgA^+^ B cells in PPs from WT mice after oral immunization (left). Quantification of the glucose dependence, mitochondrial dependence, glycolytic capacity and fatty acid and amino acid oxidation (FaaO) capacity of NP^+^CTB^+^IgM^+^ B cells and NP^+^CTB^+^IgA^+^ B cells in PPs via the SCENITH assay (right). **F** Kinetics of HIF-1α and c-Myc protein expression in WT splenic B cells cultured in the indicated class switching medium and analyzed by immunoblotting. Densitometric quantification of the immunoblotting signals was normalized to that of Actin. **G** Flow cytometry analysis of surface IgG1, IgG2b, IgG2c and IgA expression in *Hif1a* KO and control B cells after stimulation, which induced IgG1, IgG2b, IgG2c and IgA class switching, respectively (*n* = 5 per group). **H** RNA-seq analysis of the top DEGs (log2 FC > 2) in *Hif1a*-deficient B cells compared with control B cells after IgA class switching (*n* = 3 per group). **I** Top 10 enriched pathways according to the gene expression profiles of the RNA-Seq data in **H** according to the GO enrichment analysis. **J** Relative glucose consumption by *Hif1a* KO and WT control B cells after IgA class switching (*n* = 6 per group). **K** ECAR of *Hif1a* KO and WT control B cells after IgA class switching (*n* = 6 per group). The ECAR is reported as mpH units per minute. **L** Representative histograms of puromycin staining of IgA^+^B220^+^ B cells from PPs from *Hif1a* cKO and littermate control mice after incubation with various metabolic inhibitors via the SCENITH assay (left). Quantification of glucose dependence, mitochondrial dependence, glycolytic capacity and FaaO capacity of IgA^+^B220^+^ B cells in PPs from *Hif1a* cKO and littermate control mice (right). The data represent the means ± SEMs. The results are representative of three independent experiments. *p* values were calculated via an unpaired two-tailed Student’s *t* test (**E**, **G**, **J**, **K**, **L**). *p < 0.05, **p < 0.01, ****p < 0.0001. NS not significant (*p* > 0.05)

Therefore, we performed single-cell RNA sequencing (scRNA-seq) analysis of CD45^+^ lymphocytes sorted from PPs to characterize the intrinsic features of IgA^+^ B cells in vivo. Nine clusters were identified, among which clusters 0 to 6 were B-cell lineages on the basis of their expression of the *Cd19* gene, whereas clusters 7 and 8 were T-cell lineages since they expressed the *Cd3e* gene (Fig. 2A, B; Supplementary Fig. 4A). Clusters 5 and 6 expressed *Igha* and were considered IgA^+^ B cells, whereas Clusters 0 to 4 expressed the *Ighm* gene and represented IgM^+^ B cells. Notably, IgA^+^ B cells displayed an enriched glycolysis signature compared with that of IgM^+^ B cells (Fig. 2C, D), implying that a glycolytic switch occurs during IgA-producing B-cell differentiation in PPs. Moreover, pseudotemporal trajectory analysis revealed that IgA^+^ B-cell cluster 5 has higher pseudotime values than the other IgM^+^ B-cell clusters (Supplementary Fig. 4B). However, HIF-1α was not detected among the top 5 predicted transcriptional regulators in IgA^+^ B-cell clusters by single-cell regulatory network inference and clustering (SCENIC) analysis (Supplementary Fig. 4C). Nevertheless, to further address the metabolic state of IgM^+^ B cells and IgA^+^ B cells, glucose uptake activity was examined in IgM^+^ B cells and IgA^+^ B cells. Interestingly, IgA^+^ B cells displayed greater glucose transport activity than IgM^+^ B cells (Supplementary Fig. 5A), suggesting that IgA^+^ B cells preferentially use glucose metabolism. By using single-cell energetic metabolism by profiling translation-in-hibition (SCENITH) technology, in vivo analyses of IgA^+^ B cells confirmed a greater level of glucose-dependent energy production than IgM^+^ B cells (Supplementary Fig. 5B, C). Furthermore, antigen-specific NP^+^CTB^+^IgA^+^ B cells presented increased glucose-dependent energy production relative to antigen-specific NP^+^CTB^+^IgM^+^ B cells in WT mice after oral immunization with NP-OVA or CT (Fig. 2E).

Next, we characterized the kinetics of HIF-1α expression during immunoglobulin class switching in vitro (Supplementary Fig. 5D). We observed that IgA class switching led to a substantial increase in HIF-1α protein but not c-Myc (Fig. 2F), another major regulatory factor that controls glycolytic metabolism [29]. In contrast, c-Myc levels were high in B cells under IgG1, IgG2b or IgG2c class switching conditions (Fig. 2F). We also tested the effect of c-Myc inhibition on B-cell class switching in vitro. At a dose of 10 μM, the c-Myc inhibitor MYCi975 was toxic to B cells. However, MYCi975 treatment at a dose of 5 μM reduced the IgG1^+^, IgG2b^+^ and IgG2c^+^ B-cell frequencies without altering IgA class switching (Supplementary Fig. 6A, B). Interestingly, we also noted that treatment with MYCi975 (5 μM) decreased glycolytic gene expression under IgG1, IgG2b and IgG2c class switching conditions (Supplementary Fig. 6C). The in vitro class-switching capacity of *Hif1a*-deficient and WT B cells was analyzed. Sorted CD21^int^CD23^+^ FO B cells from *Hif1a* cKO and WT littermate mice were cultured under IgG1, IgG2b, IgG2c or IgA class switching conditions for 72 h. Among them, only the IgA^+^ B-cell frequency was reduced in the mutant cells (Fig. 2G; Supplementary Fig. 6D). Taken together, these results indicate that c-Myc-dependent metabolic programming may control IgG1, IgG2b or IgG2c class switching, whereas metabolic rewiring for IgA class switching appears to be dependent on HIF-1α expression.

**Fig. 3:**
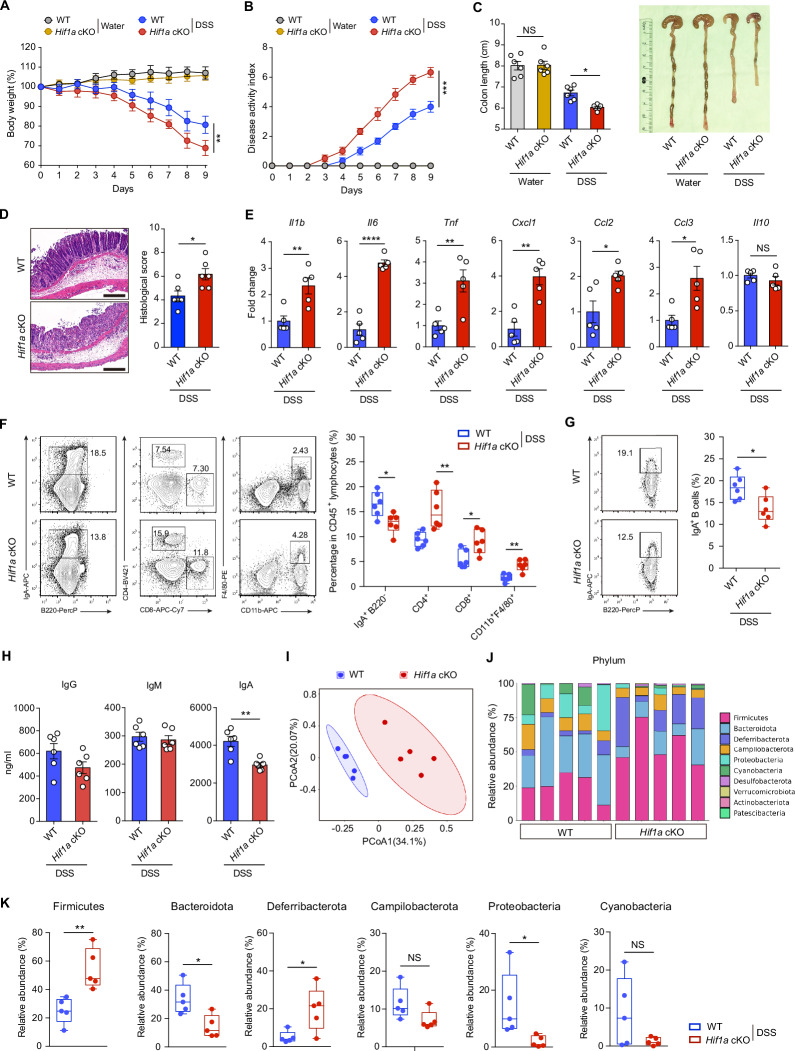
***Hif1a*** **cKO mice exhibit exacerbated inflammation in a DSS-induced colitis model**. Body weight (**A**) and disease activity index (**B**) of *Hif1a* cKO and littermate control mice that received regular drinking water or 2.5% DSS-containing water for 7 days, followed by regular drinking water for the remaining days (*n* = 5, 6 per group). **C** Lengths of the colons of *Hif1a* cKO and littermate control mice that received regular drinking water alone or 2.5% DSS-containing water for 7 days, followed by access to regular drinking water for the remaining days (*n* = 6 per group). **D** Representative H&E staining and histology scores of colon sections from *Hif1a* cKO and littermate control mice on day 9 after DSS treatment (*n* = 6 per group). Scale bars, 100 μm. **E** qPCR analysis of the relative mRNA expression of *Il1b*, *Il6*, *Tnf*, *Cxcl1*, *Ccl2*, *Ccl3* and *Il10* in colonic tissues from *Hif1a* cKO and littermate control mice on day 9 after DSS treatment (*n* = 5 per group). **F** Flow cytometry analysis of colon-infiltrated immune cells from *Hif1a* cKO and littermate control mice on day 9 after initial DSS exposure (*n* = 6 per group); the data are presented as box and whisker plots (5–95 percentiles). **G** Flow cytometry plots and quantification of B220^+^IgA^+^ B cells in PPs from *Hif1a* cKO and littermate control mice on day 9 after initial DSS exposure (*n* = 6 per group). **H** Feces were collected from *Hif1a* cKO and littermate control mice on day 9 after initial DSS exposure to detect IgG, IgM and IgA by ELISA (*n* = 6 per group). **I** PCoA of beta diversity via the Jaccard distance metric in fecal samples from WT and *Hif1a* cKO mice after initial DSS exposure. **J** Analysis of specific bacterial 16S rDNA sequences to detect commensal diversity in WT and *Hif1a* cKO mice at the phylum level after initial DSS exposure. **K** Relative abundance of the fecal microbiota in WT and *Hif1a* cKO mice at the phylum level after initial DSS exposure. The data represent the means ± SEMs. The results are representative of three independent experiments. *p* values were calculated via two-way analysis of variance with Bonferroni post hoc correction for paired data (**A**, **B**) or an unpaired two-tailed Student’s *t* test (**C**–**K**). *p < 0.05, **p < 0.01, ***p < 0.001, ****p < 0.0001. NS not significant (*p* > 0.05)

To test this hypothesis, bulk RNA-seq and quantitative real-time PCR (qPCR) were performed on *Hif1a* KO B cells and WT control B cells during IgA class switching. Interestingly, the expression of genes related to glycolysis but not oxidative phosphorylation or B-cell differentiation was reduced in the mutant cells (Fig. 2H; Supplementary Fig. 7). We also observed that the glycolytic pathway was significantly enriched according to the Gene Ontology (GO) database (Fig. 2I). Moreover, glucose consumption and extracellular acidification rate (ECAR) analyses revealed less glucose consumption and lower glycolytic activity in *Hif1a* KO B cells than in WT B cells during IgA class switching (Fig. 2J, K). In vivo analyses of *Hif1a* KO IgA^+^ B cells from PPs confirmed a low level of glucose-dependent energy production via the SCENITH assay (Fig. 3L). These results indicate that IgA production in B cells is most likely dependent on HIF-1α-mediated glycolytic metabolism.

### Exacerbated inflammation in DSS-treated *Hif1a*-deficient mice

Given that mucosal IgA plays a crucial role in colon homeostasis [15], we characterized *Hif1a* cKO mice subjected to DSS-induced colitis. The mutant mice developed more severe colitis, as indicated by rapid body weight loss, increased rectal bleeding and diarrhea (Fig. 3A, B), short colon length and marked intestinal epithelial damage (Fig. 3C, D). The molecular characterization of intestinal inflammation via qPCR revealed that *Hif1a* cKO mice presented increased proinflammatory cytokine and chemokine expression in mucosal tissues (Fig. 3E). In line with these data, the infiltration of inflammatory immune cells, including CD4^+^ T cells, CD8^+^ T cells and CD11b^+^F4/80^+^ macrophages, was increased in the colonic tissue of *Hif1a* cKO mice (Fig. 3F). In addition, IgA^+^ cell numbers and secreted IgA levels were lower in *Hif1a* cKO mice than in littermate control mice during DSS-induced colitis (Fig. 3F–H). These data suggest that HIF-1α-mediated mucosal IgA production may contribute to protection against DSS-induced colitis.

The microbiota contributes to the pathogenesis of colitis [30–32]. We next sought to investigate whether HIF-1α-mediated IgA production contributes to the balanced microbiome in the gut. 16S rRNA sequencing and Jaccard principal coordinate analyses revealed that the microbiome in the *Hif1a* cKO mouse colon clustered separately from that in the WT colon (Fig. 3I). Moreover, analysis of the relative abundance of predominant bacterial clades revealed that at the phylum level, feces from *Hif1a* cKO mice presented decreased abundance of *Bacteroidota* and increased abundance of *Firmicutes* and *Deferribaterota* (Fig. 3J, K). These findings indicate that HIF-1α expression in B cells supports proper IgA production and that the presence of mucosal IgA is a critical factor for a well-balanced microbiome in the gastrointestinal tract.

### The PHD inhibitor roxadustat ameliorates DSS-induced colitis

**Fig. 4:**
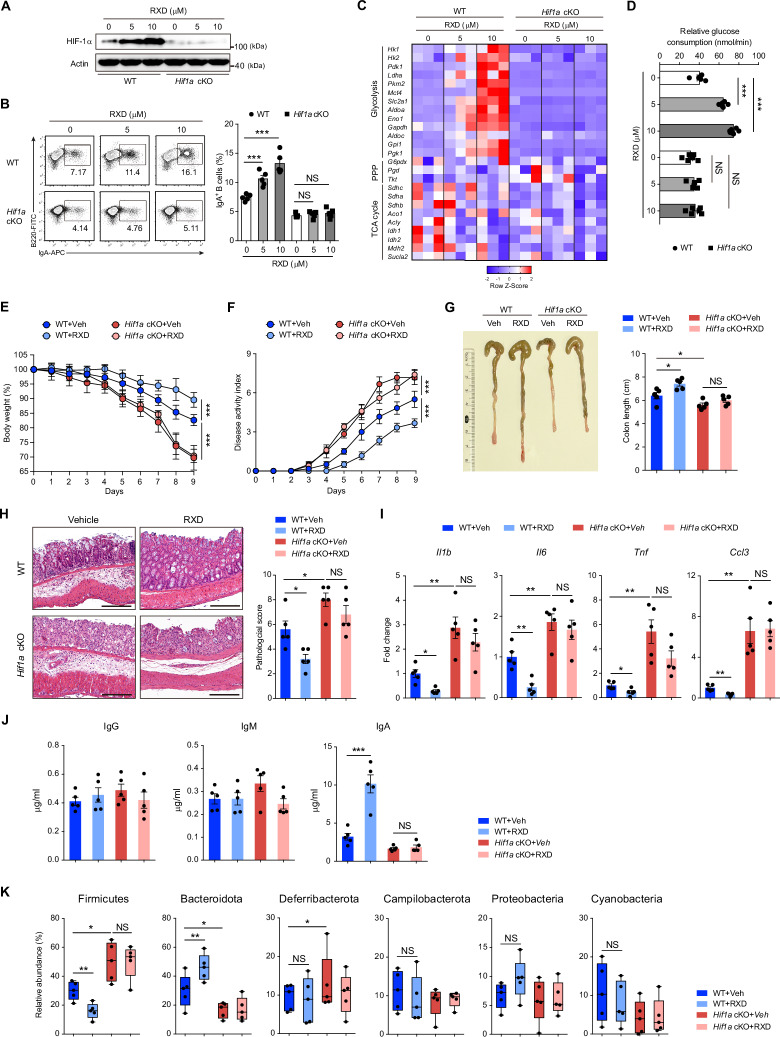
**HIF-1α stabilization by the PHD inhibitor roxadustat alleviates DSS-induced intestinal inflammation**. **A** Immunoblotting analysis of HIF-1α expression levels in roxadustat (RXD) (0, 5, 10 μM)-treated WT or *Hif1a* KO B cells 24 h after initiating IgA class switching. **B** Flow cytometry plots and quantification of IgA^+^ B cells in WT or *Hif1a* KO B cells after initiating IgA class switching supplemented with RXD (0, 5, or 10 μM) (*n* = 5 per group). **C** Heatmap displaying glycolysis-, PPP- and TCA cycle-related genes analyzed by qPCR in WT or *Hif1a* KO B cells after initiating IgA class switching supplemented with RXD (0, 5, or 10 μM) (*n* = 3 per group). **D** Relative glucose consumption by WT or *Hif1a* KO B cells after initiating IgA class switching supplemented with RXD (0, 5, or 10 μM) (*n* = 6 per group). **E,** **F** Body weight (**E**) and disease activity index (**F**) of 8-week-old *Hif1a* cKO mice and WT littermate control mice that received 2.5% DSS combined with vehicle (Veh) or RXD injection (*n* = 5 per group). **G** Colon lengths of *Hif1a* cKO mice and WT littermate control mice that received 2.5% DSS combined with vehicle or RXD injection (n = 5 per group) on day 9. **H** Representative H&E staining and histology scores of colon sections from *Hif1a* cKO mice and WT littermate control mice that received DSS combined with vehicle or RXD injection (*n* = 5 per group) on day 9. Scale bars, 100 μm. **I** qPCR analysis of *Il1b*, *Il6*, *Tnf* and *Ccl3* mRNA levels in the colon tissues of *Hif1a* cKO mice and WT littermate control mice that received DSS combined with vehicle or RXD injection (*n* = 5 per group) on day 9. **J** Feces were collected from *Hif1a* cKO mice and WT littermate control mice that received DSS combined with vehicle or RXD injection to detect IgG, IgM and IgA by ELISA (*n* = 5 per group) on day 9. **K** Relative abundance of the fecal microbiota in *Hif1a* cKO mice and WT littermate control mice at the phylum level after receiving DSS combined with vehicle or RXD injection. The data represent the means ± SEMs. The results are representative of three independent experiments. *p* values were calculated via an unpaired two-tailed Student’s *t* test (**J**), one-way ANOVA followed by Dunnett’s test (**B**, **D**, **G**, **H**–**K**) or two-way analysis of variance with Bonferroni post hoc correction for paired data (**E**, **F**). *p < 0.05, **p < 0.01, ***p < 0.001, ****p < 0.0001. NS not significant (*p* > 0.05)

We next examined whether the activation of HIF-1α signaling could enhance IgA production in B cells. To test this hypothesis directly, we used roxadustat (RXD), a small molecule that inhibits PHD activity, thereby stabilizing HIF-1α. Roxadustat is used to treat renal anemia in humans [33]. In our experimental setup, HIF-1α protein levels increased in a dose-dependent manner during IgA class switching (Fig. 4A). Notably, 5 μM or 10 μM RXD supplementation enhanced IgA class switching through HIF-1α stabilization (Fig. 4B). Gene profiling analyses and glucose consumption revealed that RXD application increased glucose consumption, glycolytic gene expression and glycolytic activity in cultured WT B cells during IgA class switching in a dose-dependent manner (Fig. 4C, D). To further assess the potential of RXD to increase IgA production in vivo, WT mice were treated with either vehicle control or RXD (10 mg/kg) for a period of nine days. The proportion of IgA^+^ cells was increased in the PPs and iLPs of the mice treated with RXD (Supplementary Fig. 8A). In accordance with the aforementioned findings, serum and fecal IgA levels were upregulated following RXD treatment (Supplementary Fig. 8B, C). Furthermore, compared with those from vehicle-treated control mice, sorted IgA^+^ B cells from RXD-treated mice presented elevated levels of glycolytic genes, including *Pkm2, Gapdh, Ldha, Mct4, Gpi1* and *Hk2* (Supplementary Fig. 8D). Collectively, these data indicate that HIF-1α stabilization by a PHD inhibitor RXD metabolically enhances IgA class switching in B cells.

As RXD enhances IgA class switching in B cells both in vitro and in vivo, we evaluated whether RXD has a potential therapeutic benefit for treating intestinal inflammation. RXD (10 mg/kg) was intraperitoneally (i.p.) injected daily into WT and *Hif1a* cKO mice subjected to DSS. We observed that targeting the PHD/HIF-1α axis with RXD decreased body weight loss and the colitis disease activity index (DAI) in WT mice but not in *Hif1a* cKO mice (Fig. 4E, F). Colon length, which represents a surrogate macroscopic indicator of colonic injury, was also improved in WT mice treated with RXD (Fig. 4G). Pathology analyses of the colon lesions revealed that RXD treatment restored the DSS-induced disruption of the crypt architecture and reduced inflammatory cell infiltration in WT mice (Fig. 4H). Given that excessive proinflammatory cytokine production is a hallmark of intestinal inflammation and IBD clinical symptoms, we measured the mRNA expression of proinflammatory cytokines and chemokines in colon tissues on day 9 after DSS exposure. RXD reduced the levels of *Il1b*, *Il6*, *Tnf* and *Ccl3* in colon tissues from WT mice (Fig. 4I). In addition, RXD treatment increased the production of fecal IgA in WT mice (Fig. 4J). Next, we compared the microbiota composition in the guts of WT mice and *Hif1a* cKO mice treated with vehicle or RXD during DSS-induced colitis. The relative abundances of two different bacterial phyla significantly changed, with *Firmicutes* being reduced and *Bacteroidota* being increased in WT mice after RXD treatment (Fig. 4K). In contrast, a similar composition of the fecal microbiota was observed in *Hif1a* cKO mice treated with vehicle or RXD. Taken together, these results demonstrate that RXD protects against DSS-induced intestinal inflammation through a HIF-1α-dependent IgA mucosal response.

### Acetyl-CoA accumulation regulates histone H3K27 acetylation at the Sα region

**Fig. 5:**
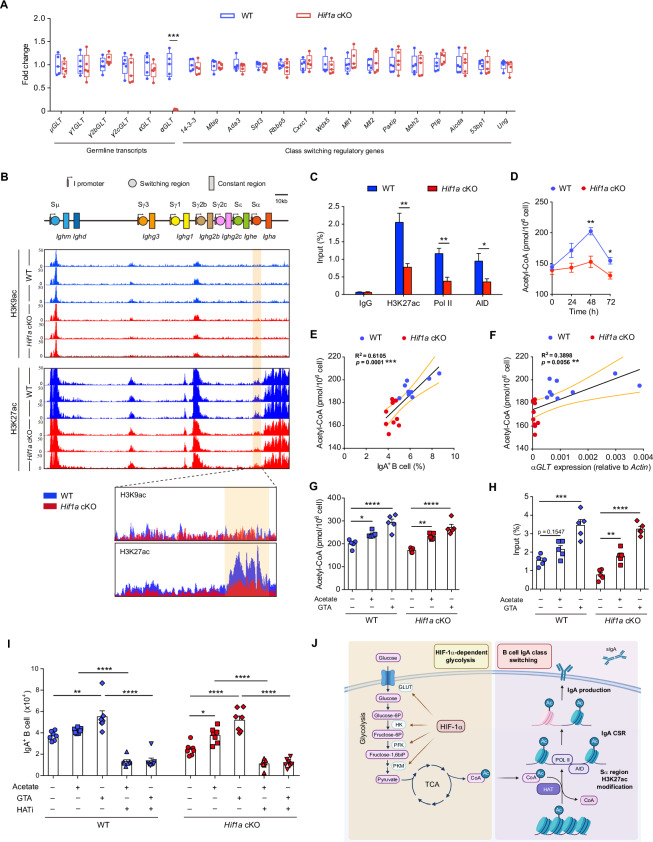
**Metabolite acetyl-CoA accumulation induced by HIF-1α regulates histone H3K27 acetylation at the Sα region, controlling B-cell IgA class switching**. **A** qPCR analysis of germline transcripts and class switching regulatory genes in *Hif1a* KO and control B cells after culture under IgA class switching conditions (*n* = 5 per group). **B** ChIP-seq (H3K9ac and H3K27ac) profiles of the *Igh* locus from *Hif1a* KO and control B cells after culture under IgA class switching conditions (*n* = 3 per group). The α class switching (Sα) region is highlighted in yellow. **C** ChIP‒qPCR assay of H3K27ac histone modification and Pol II or AID binding at the Sα region in *Hif1a* KO and control B cells after culture under IgA class switching conditions (*n* = 4 per group). **D** Kinetics of acetyl-CoA levels in *Hif1a* KO and control B cells cultured under IgA class switching conditions (*n* = 5 per group). **E** Correlation between acetyl-CoA levels and the percentage of IgA^+^ B cells in *Hif1a* KO and control B cells after culture under IgA class switching conditions (*n* = 9 per group). **F** Correlation between acetyl-CoA levels and the expression of *αGLT* in *Hif1a* KO and control B cells after culture under IgA class switching conditions (*n* = 9 per group). **G** Levels of acetyl-CoA in *Hif1a* KO and control B cells after culture under IgA class switching conditions supplemented with acetate (10 mM) or GTA treatment (5 mM) (*n* = 5 per group). **H** Histone H3K27ac levels in the Sα region in *Hif1a* KO and control B cells after culture under IgA class switching conditions supplemented with acetate (10 mM) or GTA treatment (5 mM) (*n* = 5 per group). **I** Numbers of IgA^+^ B cells in *Hif1a* KO and control B cells after culture under IgA class switching conditions with acetate (10 mM) or GTA treatment (5 mM) coupled with a HAT inhibitor (CPTH12 (10 μM)) (*n* = 7 per group). **J** Schematic picture showing the role of HIF-1α-dependent glycolytic programming in the epigenetic regulation of the Sα region during the process of IgA class switching recombination. The data represent the means ± SEMs. The results are representative of three independent experiments. *p* values were calculated via an unpaired two-tailed Student’s *t* test (**A**, **C**, **D**), Pearson’s test (**E**, **F**) or one-way ANOVA followed by Dunnett’s test (**G**–**I**). *p < 0.05, **p < 0.01, ***p < 0.001, ****p < 0.0001

To further explore the molecular mechanism that causes IgA class switching failure in *Hif1a*-deficient B cells, germline transcripts (GLTs) and class switching regulatory genes were analyzed in *Hif1a* KO and WT B cells after culture under IgA class switching condition for 48 h. Unexpectedly, the level of αGLT expression was significantly lower in *Hif1a* KO B cells than in WT control B cells, whereas other germline transcripts, such as μGLT, γ1GLT, γ2bGLT, γ2cGLT and εGLT, were unaffected (Fig. 5A). The interconnection between metabolic programming and chromatin remodeling during IgA class switching suggests that HIF-1α-dependent glycolytic flux may control epigenetic modifications at the α class switching region (Sα) in the *Igh* locus. To test our hypothesis, we examined histone acetylation (H3K9ac and H3K27ac) in *Hif1a* KO and WT B cells during IgA class switching via chromatin immunoprecipitation sequencing (ChIP-seq). Despite the comparable level of H3K9ac in both groups, a mild reduction in the level of H3K27ac was observed in the Sα region in *Hif1a* KO B cells compared with WT B cells (Fig. 5B). Moreover, no difference was detected in the Sγ1 region, Sγ2b region or Sγ2c region between *Hif1a* KO B cells and WT B cells (Supplementary Fig. 9A). Notably, this reduction in H3K27 acetylation was associated with a decrease in Pol II and AID occupancy at the Sα region in *Hif1a*-deficient B cells (Fig. 5C). Collectively, our data suggest that HIF-1α deficiency in B cells diminishes histone H3K27 acetylation and Pol II and AID occupancy at the Sα region, which is required for proper IgA class switching recombination.

We then assessed the metabolic changes occurring in *Hif1a* KO B cells during IgA class switching. By using a ^13^C_6_-glucose tracing assay, we found that HIF-1α deficiency impaired the turnover rate of carbohydrate metabolism processes and decreased the glycolytic function of B cells (Supplementary Fig. 9B, C). Interestingly, the key substrate for histone acetylation, acetyl-CoA, was decreased in *Hif1a* KO B cells during IgA class switching recombination (Fig. 5D). A positive correlation between acetyl-CoA levels and the frequency of IgA^+^ B cells or αGLT expression was observed during IgA class switching (Fig. 5E, F), suggesting that the level of acetyl-CoA might be associated with IgA class switching recombination in B cells. Thus, we sought to address whether acetyl-CoA supplementation by acetate or glyceryl triacetate (GTA) treatment could alter histone H3K27 acetylation at the Sα region. Indeed, both acetate (10 mM) and GTA (5 mM) treatments increased acetyl-CoA levels in B cells (Fig. 5G) and restored histone H3K27 acetylation at the Sα region in *Hif1a-*deficient B cells during IgA class switching (Fig. 5H). Moreover, inhibition of histone acetyltransferase (HAT) activity by the specific inhibitor CPTH2 prevented acetate- or GTA-mediated IgA production, indicating that HAT activity is essential for IgA production by acetyl-CoA supplementation in *Hif1a* KO B cells (Fig. 5I). Given the crucial role of the noncanonical NF-κB pathway and TGF-β signaling in IgA class switching, we further investigated the effects of acetate or GTA treatment on these pathways by immunoblotting analysis. The protein levels of TRAF2, TRAF3, p100 and RelB remained similar in activated B cells after acetate or GTA treatment (Supplementary Fig. 9D). Moreover, no significant difference in the expression of pSmad3 was detected among the vehicle-, acetate- or GTA-treated groups during IgA class switching (Supplementary Fig. 9D). These results suggested that the noncanonical NF-κB pathway and TGF-β signaling were not involved in the effects of acetate or GTA treatment on B-cell IgA class switching. Taken together, these results demonstrated that acetyl-CoA accumulation induced by HIF-1α-dependent glycolytic flux controls histone H3K27ac modification at the Sα region, which is set to undergo IgA class switching recombination (Fig. 5J).

### Acetyl-CoA supplementation restores IgA production in mucosal tissue and ameliorates DSS-induced colitis

**Fig. 6:**
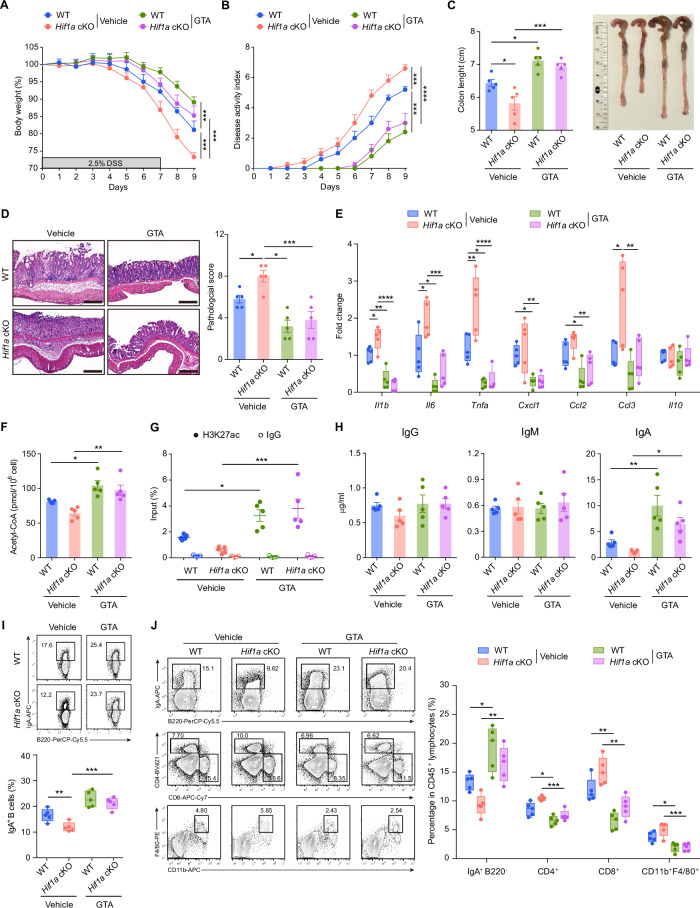
**Acetyl-CoA supplementation by GTA ameliorates DSS-induced colitis in** ***Hif1a*** **cKO mice and restores IgA production in mucosal tissue**. Body weight (**A**) and disease activity index (**B**) of DSS-exposed *Hif1a* cKO mice and littermate control mice that were treated daily with vehicle or GTA (4 g/kg) by oral administration for 9 days. Colon lengths (**C**) and representative H&E staining results coupled with the histology score of the colons (**D**) on day 9 after initial DSS exposure. Scale bars, 100 μm. **E** qPCR analysis of *Il1b*, *Il6*, *Tnf*, *Cxcl1*, *Ccl2*, *Ccl3* and *Il10* mRNA levels in colon tissues on day 9 after initial DSS exposure, represented as box and whisker plots (5–95 percentiles). **F** Acetyl-CoA levels in pooled B cells from MLNs and PPs on day 9 after initial DSS exposure. **G** ChIP‒qPCR analysis of H3K27ac levels in the Sα region in pooled B cells from MLNs and PPs on day 9 after initial DSS exposure. **H** Feces were collected to detect IgG, IgM and IgA by ELISA on day 9 after initial DSS exposure. **I** Flow cytometry plots and quantification of B220^+^IgA^+^ B cells in PPs on day 9 after initial DSS exposure. **J** Flow cytometry analyses of colon-infiltrated immune cells on day 9 after initial DSS exposure, represented as box and whisker plots (5th–95th percentiles). The data are presented as representative plots and summary graphs of quantified percentages. The data represent the means ± SEMs. The results are representative of three independent experiments. *p* values were calculated via two-way analysis of variance with Bonferroni post hoc correction for paired data (**A**, **B**) or one-way ANOVA followed by Dunnett’s test (**C**–**J**). *p < 0.05, **p < 0.01, ***p < 0.001, ****p < 0.0001

GTA, a compound for acetyl-CoA supplementation in vivo [34], has the potential to promote IgA production. To test this hypothesis, the mice were orally administered GTA (4 g/kg) or vehicle once daily. We observed that C57BL/6 WT mice treated with GTA presented increased levels of acetyl-CoA, increased quantities of IgA^+^ B cells in PPs, and secretory IgA in feces (Supplementary Fig. 9E–H). We next tested the impact of GTA treatment on a DSS-induced colitis model. Notably, treatment with GTA alleviated the symptoms of colitis in both *Hif1a* cKO mice and control mice after DSS exposure, as indicated by improvements in body weight, colon length and histology (Fig. 6A–D). These changes were accompanied by reduced inflammatory cytokine or chemokine expression in the colon (Fig. 6E). Additionally, GTA treatment increased acetyl-CoA levels and downstream histone H3K27 acetylation at the Sα region in B cells from *Hif1a* cKO mice during DSS-induced colitis (Fig. 6F, G). Consistent with the improved colitis clinical symptoms, GTA treatment decreased the number of infiltrating T cells and macrophages in the colon and increased the number of IgA^+^ B cells and IgA^+^ plasma cells as well as the secretion of IgA in the stool of *Hif1a* cKO mice (Fig. 6H–J). Together, these data indicate that GTA treatment in vivo protects against the aggravated colitis phenotype of *Hif1a* cKO mice, most likely through enhanced IgA-producing B-cell differentiation.

## Discussion

Germinal center (GC) B cells exhibit high metabolic activity, consume elevated levels of glucose and express genes associated with glycolysis, OXPHOS and the electron chain [8, 35, 36]. Nevertheless, which metabolic pathways are responsible for coordinating the differentiation and function of GC B cells in vivo is incompletely understood. Although studies reporting that hypoxic microenvironments in GC areas are crucial, they have focused solely on the impact of low oxygen on in vitro cell culture or sustained HIF-1α stabilization in vivo by VHL deletion on B-cell activation and differentiation [7, 37]. The deletion of VHL in B cells has been demonstrated to adversely affect immature B-cell differentiation in the bone marrow [37]. Moreover, B cells are unable to switch to IgG2c and exhibit abnormal proliferation under hypoxic conditions [7]. Here, we show that HIF-1α specifically regulates IgA-producing B-cell differentiation by increasing glycolytic metabolism. This can be seen in the primary regulation of glucose consumption and glycolysis by HIF-1α. In line with previous research that emphasized the importance of metabolic reprogramming for IgA-producing cell differentiation in GALT [38, 39], we demonstrated that a glycolytic shift governed by HIF-1α is critical for efficient IgA class switching in B cells.

However, accumulating evidence indicates that B cells display dynamic metabolic changes to meet fluctuating energetic demands throughout the different phases of the humoral response [40, 41]. Our data suggest that the protein levels of the critical transcription factors c-Myc and HIF-1α in glucose metabolism [42] are differentially affected during IgG1, IgG2b, IgG2c and IgA class switching. A study using c-Myc mutant mice revealed that c-Myc is necessary for the generation of fully differentiated antibody-secreting cells (ASCs) and IgG1 class switching [43]. Our findings suggest that c-Myc-dependent metabolism may serve as an important regulator of IgG1, IgG2b and IgG2c class switching, whereas HIF-1α-dependent metabolic programming is unique for IgA class switching. The metabolic pathways of glycolysis and OXPHOS are recognized as regulators of dynamic chromatin modifications via cellular intermediary metabolites, e.g., acetyl-CoA, S-adenosylmethionine (SAM), flavin adenine dinucleotide (FAD) and α-ketoglutarate (αKG). These metabolites act as substrates and/or cofactors to affect the enzymatic activity of chromatin modifiers [44, 45]. Previously published data and our study indicate the essential role of histone epigenetic modifications in directing the CSR machinery toward the upstream donor and downstream acceptor S regions [46]. The absence of HIF-1α signaling in B cells resulted in decreased acetyl-CoA levels, leading to the impairment of H3K27ac modification at the Sα region. Consequently, a connection between HIF-1α-induced acetyl-CoA accumulation and chromatin modification is vital in IgA class switching.

Interactions between B cells and DCs or T cells in PPs facilitate proliferation, somatic hypermutation and class-switching processes in the germinal center [47]. Accordingly, our scRNA-seq data demonstrated that, compared with IgM^+^ naïve B cells, class-switched IgA^+^ B cells exhibited an increased capacity for proliferation and glycolysis. Notably, there was no significant difference in the proportion of proliferating *Hif1a*-deficient IgA^+^ B cells compared with that of WT IgA^+^ B cells. These findings suggest that HIF-1α-dependent glycolysis is dispensable for IgA^+^ B-cell proliferation. In addition to glucose, glutamine and lipids are essential nutrients for cellular growth, serving not only anabolic processes but also energy metabolism [48, 49]. A distinct pattern of energy metabolism may be necessary for IgA^+^ B-cell class switching and IgA^+^ B-cell proliferation. Nevertheless, glutamine or lipid metabolism may compensate for impaired glycolysis during the proliferation of *Hif1a*-deficient IgA^+^ B cells.

Given that IgA deficiency predisposes individuals to IBD [50] and exacerbates the severity of DSS-induced colitis in mice [18], it has been proposed that targeting the PHD/HIF-1α axis with the PHD inhibitor roxadustat, the first HIF stabilizer approved for the clinical treatment of renal anemia [33], may modulate IgA responses and alleviate experimental intestinal disease. Our study reveals a new protective function of roxadustat in DSS-induced experimental colitis. Roxadustat administration significantly enhanced IgA production and mitigated intestinal inflammation. Notably, studies have indicated that roxadustat alleviates the disruption of epithelial tight junctions and inhibits macrophage infiltration in chronic kidney disease [51, 52]. Therefore, more studies are needed to elucidate the effects of roxadustat on epithelial and other immune cell populations in addition to B cells. In both experimental autoimmune encephalomyelitis (EAE) and collagen-induced arthritis (CIA), the differentiation of IL-10^+^ regulatory B cells is impaired as a result of HIF-1α deficiency [25]. Nevertheless, our findings revealed no notable difference in IL-10 expression between *Hif1a* cKO mice and WT mice following DSS exposure. This observation implies that additional innate immune cells, such as macrophages and dendritic cells, may also influence the IL-10 level during DSS-induced acute intestinal inflammation. In addition, this finding is consistent with a previous report that B cells mitigate colitis through an IL-10-independent pathway [53]. Previous studies have demonstrated that the IgE response also plays an important role in mucosal inflammation [54, 55]. However, we did not detect a significant difference in IgE levels between *Hif1a* cKO mice and WT littermate control mice, suggesting that IgE-producing B cells might not be involved in exacerbated inflammation in DSS-treated *Hif1a*-deficient mice.

IgA modulates the intestinal microbiota, and IgA deficiency often leads to gut dysbiosis [24]. An earlier study reported that MZB1 (pERp1) deficiency in B cells aggravated dysbiosis during the course of DSS-induced colitis, with impaired IgA secretion in the gut and a dramatic reduction in *Bacteroidota* [22]. In line with these findings, a modified microbiome was observed in the stool of *Hif1a* cKO mice following DSS treatment, suggesting that IgA-mediated microbial homeostasis in the intestinal mucosa may influence the symptoms of DSS-induced colitis.

In summary, we provide a novel molecular mechanism for the regulation of mucosal IgA production by HIF-1α. HIF-1α stabilization by the PHD inhibitor roxadustat effectively enhanced glycolysis, leading to increased IgA production and reduced colonic inflammation in DSS-induced colitis. By modulating histone H3K27 acetylation at the Sα region, HIF-1α-mediated glycolytic flux controls IgA class switching recombination and IgA-producing cell differentiation. Moreover, pharmacological supplementation of acetyl-CoA by GTA reversed IgA production in *Hif1a* cKO B cells and protected against intestinal inflammation.

## Materials and methods

### Animal experiments

To generate B-cell-specific *Hif1a* knockout mice, *Hif1a*^f/f^ mice were crossed with *Mb1*-cre mice. *Hif1a*^f/f^ mice, *Mb1*-cre mice and Ig-α^-/-^ mice were generated as previously described [56–59]. The mice were bred and maintained on a C57BL/6 background, and *Hif1a*^f/+^ cre-positive littermates or *Hif1a*^f/f^ cre-negative littermates were used as WT control mice. For the adoptive transfer experiment, sorted CD21^int^CD23^+^ FO B cells (2 × 10^7^ cells per mouse) from *Hif1a*-deficient or littermate control mice were transferred intravenously into B-cell-deficient (Ig-α^-/-^) recipient mice (bKO mice). For the oral immunization model, the mice received sodium bicarbonate solution to neutralize stomach acid and were orally immunized with 1 mg of OVA (Sigma) or 1 mg of NP-OVA (Biosearch Technologies) plus 10 μg of CT (List Biological Laboratories) on days 0, 7 and 14. One week after the final immunization, fecal samples and mononuclear cells from the intestinal lamina propria (iLP) were collected for enumeration of OVA- or CTB-specific antibody responses via ELISA and ELISPOT. For the experimental colitis model, the mice received 2.5% DSS (MP Biomedicals) for 7 days, followed by normal drinking water for the remaining days. The medium was changed every 2 days. The mice were observed daily, and the disease activity index (DAI) was calculated according to a published protocol [60]. Briefly, the DAI is the combined score of weight loss, stool consistency, and body posture. The scores were evaluated as follows: weight loss, 0 (no loss), 1 (5 to 10%), 2 (10 to 15%), and 3 (>15%); stool consistency, 0 (normal), 1 (mildly loose stool), 2 (loose stool), and 3 (diarrhea and bloody stools); and body posture, 0 (smooth fur without hunchback), 1 (mild fur and hunchback), 2 (moderate fur and hunchback), and 3 (severe fur and heavy hunchback). For preventive treatment, the mice were intraperitoneally (i.p.) injected daily with roxadustat (10 mg/kg) [61], starting from day 0 (DSS induction) until the end of the experiment. For acetyl-CoA supplementation in the DSS model, the mice were gavaged daily with vehicle (water) or glyceryl triacetate (GTA) (4 g/kg) [62] from day 0 (DSS induction) until the end of the experiment. The animals were kept in a specific pathogen-free facility, and the animal experiments were approved by the local ethics committee of Regierung von Mittelfranken (55.2-2532-2-715).

### Cell isolation and flow cytometry

Single-cell suspensions were prepared from the bone marrow (BM), spleen (SP), mesenteric lymph nodes (MLNs), Peyer’s patches (PPs) or intestinal lamina propria (iLP). To isolate mononuclear cells from PPs, we stirred the tissues in RPMI-1640 medium containing 2% fetal calf serum plus 0.5 mg/ml collagenase (Wako, Osaka, Japan). To isolate mononuclear cells from the iLP, the PPs were carefully removed, and the remaining intestines were opened longitudinally, washed with RPMI-1640, cut into 2 cm pieces and stirred for 20 min at 37 °C in RPMI-1640 containing 0.5 mM EDTA and 2% fetal calf serum to remove epithelial cells and intraepithelial lymphocytes. The tissues were then stirred three times in 0.5 mg/ml collagenase for 20 min before being subjected to discontinuous Percoll gradient centrifugation (40 and 75%). The cells were Fc-blocked (BioLegend) and stained with surface antibodies for 30 min at 4°C. Analyses of the expression of cell surface molecules at the single-cell level were performed via flow cytometry with a Cytoflex flow cytometer (Beckman Coulter). Dead cells were detected via a LIVE/DEAD Fixable Violet Dead Cell Stain Kit (Life Technologies) before cell surface staining. For intracellular staining, B cells were fixed and permeabilized with the Foxp3/Transcription Factor Staining Buffer Set (eBioscience) according to the manufacturer’s instructions. With multiple-channel staining, the FMO control and isotype IgG are used for population gating and threshold setting. All flow cytometry experiments were gated on viable, single lymphocytes, and the data were analyzed with FlowJo software (Treestar) or Cytexpert (Beckman). Antibody information can be found in Supplementary Table 1.

### Quantitative real-time PCR

Tissue RNA was extracted via the RNeasy Mini Kit (Qiagen) or the PeqGold Total RNA Kit (Peqlab) according to the manufacturer’s instructions. A total of 2 μg of RNA was transcribed into complementary DNA (cDNA) via the High-Capacity cDNA Reverse Transcription Kit (Life Technologies). The transcribed cDNA was used for qPCR as described previously with the primers listed in Supplementary Table 2.

### FO B-cell sorting and class switching recombination culture

CD21^int^CD23^+^ FO B cells were sorted from splenocytes via a MoFlo cell sorter (DAKO instrument). A cell purity of 98–99% was generally achieved. Next, sorted B cells were resuspended in FCS-IMDM in 24-well plates and stimulated with LPS (5 μg/ml) or F(ab’)_2_ anti-IgM (1 μg/ml) plus anti-CD40 (1 μg/ml), BAFF (10 ng/ml), IL-4 (10 ng/ml) for CSR to IgG1; LPS (5 μg/ml) or F(ab’)_2_ anti-IgM (1 μg/ml) plus anti-CD40 (1 μg/ml), BAFF (10 ng/ml) for CSR to IgG2b; LPS (5 μg/ml) or F(ab’)_2_ anti-IgM (1 μg/ml) plus anti-CD40 (1 μg/ml), BAFF (10 ng/ml), IFN-γ (10 ng/ml) for CSR to IgG2c; LPS (5 μg/ml) or F(ab’)_2_ anti-IgM (1 μg/ml) plus anti-CD40 (1 μg/ml), BAFF (10 ng/ml), IL-5 (10 ng/ml), TGF-β (10 ng/ml) for CSR to IgA. The PHD inhibitor roxadustat (5, 10 μM), acetate (10 mM), GTA (5 mM) or the HAT inhibitor CPTH2 (10 μM) were added to the cultures. B cells were analyzed for surface expression of Ig after 72 h.

### ELISA and ELISPOT assay

Enzyme-linked immunosorbent assay (ELISA) and enzyme-linked immunosorbent spot (ELISPOT) were performed as described previously [63, 64]. To measure OVA- or CTB-specific Ig levels in fecal extracts or serum, the plates were coated with 1 mg/ml OVA or 2 μg/ml CTB in PBS and then blocked for 1 h at room temperature with 200 μl of PBS containing 2% (w/v) FCS. After extensive washing of the plates with PBS containing 0.05% Tween 20, serial sample dilutions were added for 2 h of incubation at room temperature. The samples were then incubated for 1 h at room temperature with optimally diluted HRP-conjugated goat anti-mouse Ig (SouthernBiotech). After sample washing, the color reaction was developed at room temperature with 1,2-benzylenediamine, and the optical density (OD) at 492 nm was measured. The ELISPOT assay was used to enumerate Ig-producing AFCs in the iLP. Briefly, various concentrations of mononuclear cells were cultured at 37 °C for 4 h in 96-well nitrocellulose membrane plates (Millipore) coated with 1 mg/ml OVA or 2 μg/ml CTB in PBS. After the plates were vigorously washed with PBS and PBS containing 0.05% Tween 20, HRP-conjugated goat anti-mouse Ig was added; the plates were then incubated overnight at 4 °C. Spots of ASCs were developed with TMB substrate (Mabtech).

### Acetyl-CoA concentration measurement

For acetyl-CoA measurement, cultured or enriched B cells were washed with cold PBS, harvested in RIPA buffer, and sonicated. The cellular or tissue protein was precipitated with PCA. After centrifugation, the supernatant was recovered and neutralized by the addition of potassium bicarbonate until the pH of the sample was in the range of 6--8, and the precipitates were removed via centrifugation. The supernatant was used to determine the acetyl-CoA concentration in triplicate via an acetyl-CoA assay kit (Sigma MAK039) according to the manufacturer’s instructions.

### Western blot analysis

Cultured B cells were homogenized in extraction buffer (8 M urea, 10% glycerol, 1% SDS, 10 mM Tris-HCl pH 6.8, complete protease inhibitor (Roche), and 1 mM sodium vanadate). Total cell lysates were resolved via 10% SDS‒PAGE and transferred to nitrocellulose membranes (Bio-Rad). The following primary antibodies were used: HIF-1α antibody (1:1000, 10006421; Cayman), c-Myc antibody (1:1000, 5605; Cell Signaling), TRAF2 (1:1000, 4712T; Cell Signaling), TRAF3 (1:1000, 4729T; Cell Signaling), NF-κB2 p100/p52 (1:1000, 4882T; Cell Signaling), RelB (1:1000, 4922T; Cell Signaling), pSmad3 (1:500, 9520T; Cell Signaling), Histone H3 (1:1000, EPR17785; Cell Signaling) and β-actin antibody (1:2000, A2066; Sigma). The western blot bands were quantified via ImageJ software.

### Extracellular flux assay and SCENITH assay

For metabolic analysis, pooled B cells enriched with MLNs and PPs and glycolytic metabolism were measured with a Seahorse Glycolysis Stress Test Kit according to the manufacturer’s instructions. The extracellular acidification rate (ECAR) was measured with a Seahorse XFe96 Analyzer (Agilent), and the values were normalized to the protein concentration and detected with a DC protein assay (Bio-Rad). The data were analyzed with the Seahorse XF Report Generator for Glyco Stress Test.

The SCENITH assay was performed as previously described [65]. Briefly, 0.5 ×10^6^ single PPs were incubated with 2DG (final concentration: 100 mM) for 10 min, followed by oligomycin (final concentration: 1 μM) for 5 min. The medium was supplemented with 10 μg/ml puromycin, and the plates were incubated for 0.5 h at 37 °C. Afterwards, the cells were washed with cold FACS buffer and detached. Next, the cells were blocked with anti-CD16/32 for 10 min at 4 °C and subsequently stained with IgA and B220 for 30 min at 4 °C. The assay strategy is illustrated in Supplementary Fig. 4B. The following intracellular staining was carried out using the intracellular Foxp3 Fixation/Permeabilization solution (eBioscience, Thermo Fisher), and the staining with puromycin was carried out with a directly labeled anti-puromycin antibody-FITC (Millipore). The percentages of glucose dependence, mitochondrial dependence, glycolytic capacity and FAO/AAO (fatty acid oxidation and amino acid oxidation) were measured via the gMFI values of puromycin-FITC from cells treated with 2-DG, oligomycin, or 2-DG and oligomycin.

### Single-cell RNA sequencing and data analysis

PPs were excised, and single-cell suspensions were stained for CD45 (Biolegend, clone 30-F11, 1:100) and LIVE/DEAD (Life Technologies, Fixable Dead Cell Stain) and sorted into CD45^+^ cells. Sorted CD45^+^ lymphocytes were subjected to 10x Chromium Single Cell 3’ Solution v2 library preparation according to the manufacturer’s instructions. Library sequencing was performed on an Illumina HiSeq 2500 sequencer to a depth of 200 million each. Reads were converted to fastq format via mkfastq from cellranger 2.1.0 (10× Genomics) and aligned to the mouse reference genome (mm10, Ensembl annotation release 91). The alignment was performed via the count command from cellranger 2.1.0 (10× Genomics). Primary analysis, quality control filtering, clustering, identification of cluster markers and visualization of gene expression were performed via the Seurat (3.1.4) package for R. Low-quality cells were removed by requiring each cell to have more than 1000 UMIs and expressing fewer than 15% mitochondrial and more than 15% ribosomal genes. In total, 610 single cells (85.1% fraction reads in cells) with a median read coverage of 247482 and a median gene coverage of 2227 passed the quality control tests. Pseudotime analysis of the B-cell lineage (clusters 0--6) was performed via the Bioconductor package Monocle 3.0.2. Single-cell regulatory network inference and clustering (SCENIC) analysis was performed on the high- and low-biomarker expression groups via the “SCENIC” package in R to identify the key transcription factors.

### RNA sequencing and data analysis

RNA was isolated via an RNeasy kit (QIAGEN), and RNA-seq was performed by Novogene (London, UK). For analysis of coding region gene expression on average, 40 million reads were obtained per sample. For analysis of noncoding regions, on average, 100 million reads were obtained per sample. The raw reads in fastq format were then processed through Novogene in-house Perl scripts to obtain clean reads by removing reads containing adapters, reads containing poly-N and low-quality reads from the raw data. The index of the mm10 mouse was built via Bowtie v2.2.3, and paired-end clean reads were aligned to the reference genome via TopHat v2.0.12. HTSeq v0.6.1 was used to count the number of reads mapped to each gene. The fragments per kilobase of transcript per million mapped reads (FPKM) value of each gene was calculated on the basis of the length of the gene and the number of reads mapped to that gene. Differential expression analysis was performed via the DESeq R package (1.18.0). The *p* values were adjusted via Benjamini and Hochberg’s method. Genes with an adjusted *p* value < 0.05 were considered differentially expressed.

### ChIP-seq and ChIP‒qPCR analysis

ChIP experiments were performed via a ChIP-IT express kit (Active Motif) according to the manufacturer’s instructions. Briefly, cultured B cells were fixed with 1% formaldehyde for 15 min. The cell pellet was resuspended in ChIP lysis buffer, and the lysate was sonicated to shear the DNA to an average fragment size of 200–1000 bp. Chromatins (20 μg) were incubated with anti-H3K27ac (ab4729, Abcam), anti-H3K9ac (06–942, Upstate), anti-Pol II (C15100055, Diagenode), anti-AID (39–2500, Thermo Fisher) or control IgG (2729, Cell Signaling) antibodies overnight at 4 °C. Next, the genomic DNA was eluted, decrosslinked overnight and purified. For ChIP-seq, the resulting libraries were sequenced on the Illumina HiSeq 2500 platform configured for 50 bp paired-end reads. The ChIP-seq reads were aligned to the mouse (mm9) genomes via Bowtie v2.2.3. Peaks were called via MACS2, and both bigwig (signal) and bed (peak calls) files were visualized via the integrated genome browser (IGB). For ChIP‒qPCR, the presence of protein binding was assessed via the primers listed in Supplementary Table 2.

### Statistical analysis

GraphPad Prism software 8.0 was used for all the statistical analyses. Data normality was analyzed by either the Shapiro‒Wilk test or the Kolmogorov‒Smirnov test (*P* > 0.1 for all groups). Statistical evaluations of two group comparisons were performed via two-tailed Student’s *t* tests. Statistical evaluations of experiments with more than two groups were performed via one-way analysis of variance (ANOVA) or two-way ANOVA. Correlations were performed via Pearson’s test. A *P* value of less than 0.05 was considered statistically significant.

## Supplementary information

Supplemental information

## Data Availability

The bulk RNA-seq data produced in this study were deposited in a public database (GEO with the accession code GSE228365). The ChIP-seq data produced in this study were deposited in a public database (GEO with the accession code GSE228366). The scRNA-seq data produced in this study were deposited in a public database (BioProject ID: PRJNA949957 in NCBI).
