# Supplementary material for: Metabolic rewiring controlled by HIF-1α tunes IgA-producing B-cell differentiation and intestinal inflammation
Source: Cell Mol Immunol. 2024 Nov 14;22(1):54–67. doi: 10.1038/s41423-024-01233-y (PMC11686098; doi:10.1038/s41423-024-01233-y)
Supplement: Supplementary file 1 — Supplemental information [file 41423_2024_1233_MOESM1_ESM.pdf]

Supplementary material and methods

Cell survival analysis

For cell survival assays, isolated lymphocytes or cultured B cells treated with vehicle or c-Myc inhibitor (MYCi975 (1, 5, 10 μM)) were stained with Zombie Aquac (Biolegend) and Annexin V (ebioscience). Cells were analyzed by flow cytometry.

Immunofluorescence (IF) staining, Hematoxylin and eosin (H&E) staining

After sacrifice, colon tissues were carefully dissected and fixed overnight in 4% paraformaldehyde. Serial paraffin sections (5 μm) were stained for H&E using H&E staining kit (Carl Roth). For IF staining, sections were incubated overnight at 4°C with primary antibodies as follows: anti-HIF-1α antibody (1:50, Sigma) and Alexa-fluor-647 conjugated anti-GL7 antibody (1:100, Biolegend) followed by 1 h incubation with Alexa-fluor-488 conjugated secondary antibodies (1:200, VECTOR). Samples were then mounted by antifade mounting medium with DAPI (VECTOR). Fluorescence images were acquired with a Zeiss confocal microscope.

In vivo EdU proliferation assay

For cell proliferation measurements in vivo, mice were injected intravenously with 2 mg of the nucleoside analog 5-ethynyl-2-deoxyuridine (EdU) (Thermofisher) in PBS. After 2.5 h, MLN or PPs were dissected and cells were then stained for surface antigens as described, followed by EdU detection using Click-iT EdU Alexa Fluor 488 Flow Cytometry Assay Kit (Thermofisher) according to manufacturer's protocol.

Glucose Uptake Cell-Based Assay

To measure glucose uptake, cells were resuspended in PBS supplemented with 300 μmol/L 2-deoxy-2-[(7-nitro-2,1,3-benzoxadiazol-4-yl)amino]-D-glucose (2-NBDG; Cayman Chemical) for 10 minutes. After a single wash, cells were analyzed by flow cytometry.

Mass Spectrometry

Activated B cells cultured in IgA class switching condition following PBS washing and re-cultured in glucose free RPMI-1640 containing 10% dialyzed FBS and uniformly labeled [$^{13}C_6$]-Glucose (Sigma) for 2 h. Next, B cells were lysed in 1 mL 80% MeOH containing reference standards. The mixtures were vortexed, centrifuges (5 min, 16,000 rpm, 4°C) and separated into two aliquots (350 μL) for further sample preparation. Furthermore, a pooled sample was prepared with aliquots (230 μL) taken from each sample. This pooled mix was split into aliquots of 350 μL, which represented quality controls (QC) and followed the same SOP as the individual samples.

The samples and QCs were dried under nitrogen at room temperature and stored at -80°C until metabolomics analysis. Prior to the analysis, the samples were reconstituted in 200 μL eluent containing internal standards, vortexed rigorously and transferred to HPLC insert vials. The samples were analyzed using an ultra-high-performance liquid chromatograph (Ultimate 3000, Thermo Fisher Scientific) hyphenated to a high-resolution mass spectrometer (QExactive Orbitrap Focus, Thermo Fisher Scientific). All samples were analyzed in both, hydrophilic interaction chromatography (HILIC) and reverse phase chromatography. For HILIC, an ACQUITY UPLC BEH Amide column (1.7 μm, 2.1 x 100 mm, Waters) was installed. The mobile phase consisted of eluent A (10 mM ammonium formate, 0.1% formic acid; FA) and eluent B (10 mM ammonium formate, 95% acetonitrile, 0.1% FA). The gradient program started with 100%B (0-2 min), which was decreased to 30% over 12 min. This concentration was hold (14-16.5 min) and returned to the starting conditions over 1 min (17.5 min). The system was equilibrated for 10 min. For RP, an ACQUITY UPLC BEH C18 column (1.7 μm, 2.1 x 100 mm, Waters) was installed. Chromatographic separation was achieved using a gradient elution, whereby eluent A consisted of water with 0.1% (vol/vol) FA and eluent B of MeOH with 0.1% (vol/vol) FA. The gradient program started with 10% B. After injection, the percentage of B increased from 10-98% over 9 min. From 9 min to 11 min the B was maintained at 98%. Finally, the starting conditions (10%B) were reconditioned from 11 min to 11.50 min.

Conditions were maintained till 15 min to re-equilibrate the column. For both, HILIC and RP, the flow rate was 0.35 mL/min, the column heater was maintained at 50°C and the autosampler temperature was 4°C. The injection volume was 2 μL. The mass spectrometer was operated in full MS/dd-MS2 (confirmatory) mode with a resolution of 17,500, a scan range of 70-800 and an automatic gain control target of 1e6. Sheath gas, aux gas and sweep gas flow rated were set to 60, 20, and 0, respectively. The spray voltage of the HESI source was 4.00 kV. All samples were analyzed in both, positive and negative mode for ionization.

Full scan MS data was centroided using vendor software, converted to mzXML format, and further analyzed using customized open-source software (El-Maven). Compounds were identified by m/z and retention time: expected m/z of de-protonated species was computed based on exact monoisotopic mass, and retention times were matched to those of previously analyzed pure standards and concurrently analyzed control samples. Peak intensities were calculated as the average of the three scans around the peak apex (‘‘AreaTop’’).

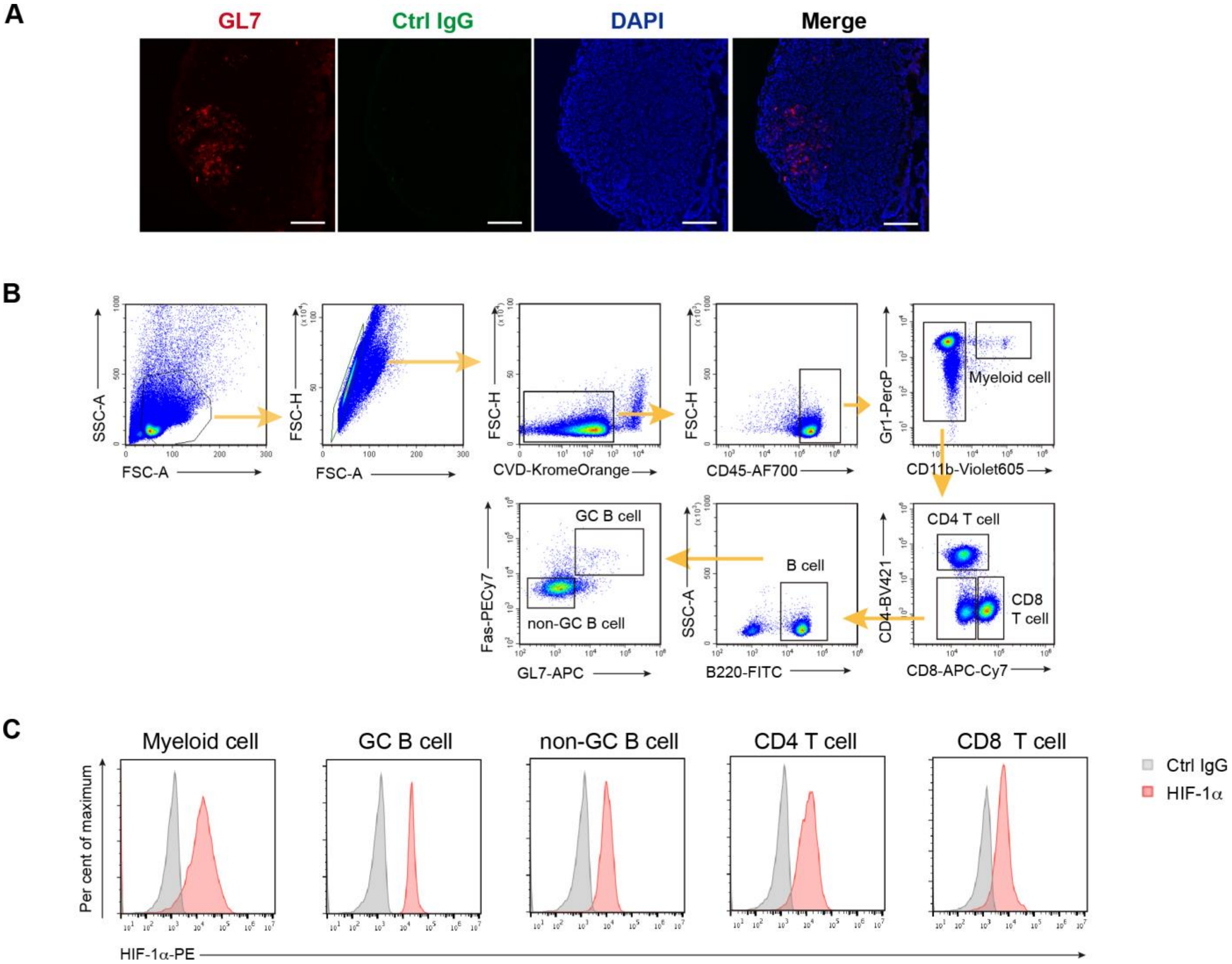

Supplementary figure 1. Analysis of HIF-1α expression in MLN or PPs by immunofluorescence and flow cytometry.

A Representative immunofluorescence microscopy images of PPs sections from 8-week-old WT mice (Control antibody, green; GL7, red, DAPI, blue). Scale bars, 100 μm. **B, C** The flow cytometry gating strategies (**B**) and flow cytometry analysis of HIF-1α expression in lymphocyte subpopulations from PPs (**C**). Results are representative of three independent experiments.

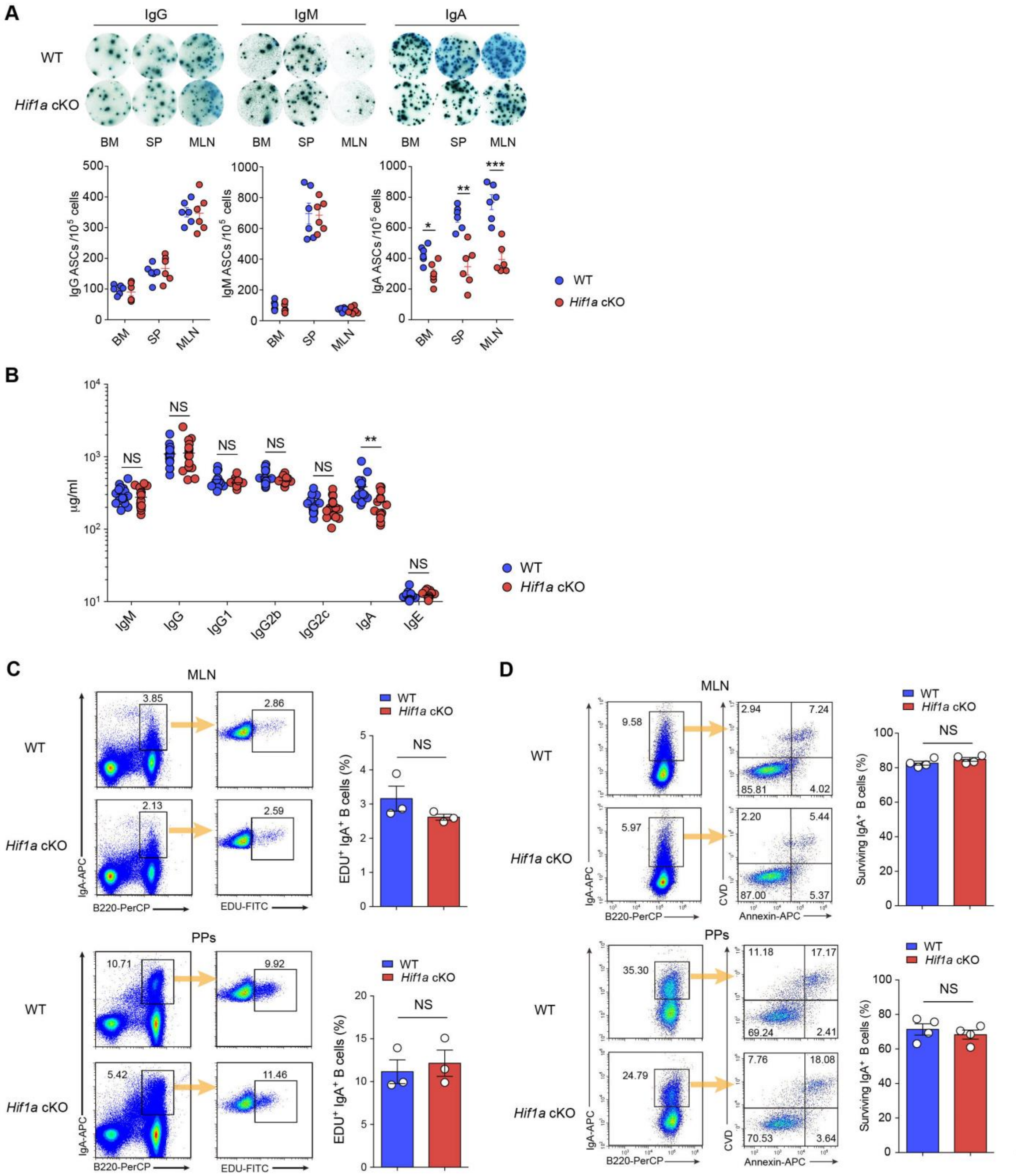

Supplementary figure 2. Antibody production, cell proliferation and survival of IgA⁺ B cells from *Hif1a*-deficient mice and WT control mice.

A ELISPOT analysis and quantification of IgG, IgM and IgA ASCs in BM, SP and MLN from WT and *Hif1a* cKO mice (n=6 per group). **B** IgM, IgG, IgG1, IgG2b, IgG2c, IgA and IgE levels in serum from WT and *Hif1a* cKO mice measured by ELISA (n=16 per group). **C** Representative plots and frequencies of ($EDU^{+}IgA^{+}$) B cell in MLN and PPs from *Hif1a* cKO (n=3) and littermate control mice (n=3). **D** Cell survival assay of $IgA^{+}B220^{+}$ B cells from *Hif1a*-deficient mice and WT control mice by Annexin-V and cell viability dye (CVD) staining (n=4 per group). Data are shown as mean ± SEM.

Results are representative of three independent experiments. p values were calculated via an unpaired two-tailed Student's t-test. $*p < 0.05$, $**p < 0.01$, $***p < 0.001$. NS, not significant ($p > 0.05$).

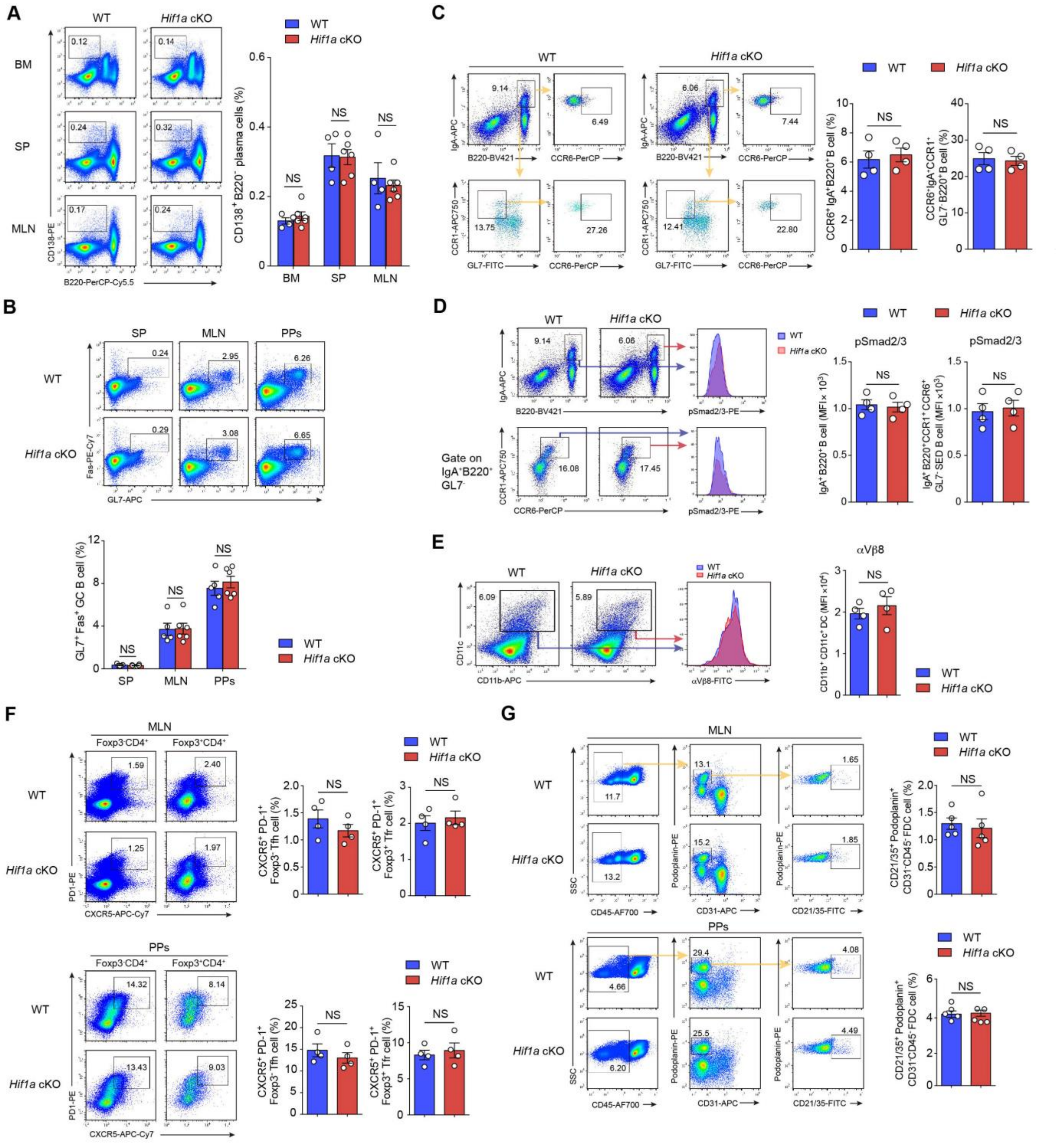

Supplementary figure 3. Plasma cell, GC B cell, Tfh cell, Tfr cell and FDC populations analyses in *Hif1a* cKO mice.

A Representative plots and frequencies of (B220$^-$CD138$^+$) plasma cell in BM, SP and MLN from *Hif1a* cKO (n=6) and littermate control mice (n=4). **B** Representative plots and frequencies of (GL7$^+$Fas$^+$) GC B cell in SP, MLN and PPs from *Hif1a* cKO (n=6) and littermate control mice (n=5). **C** Representative plots and frequencies of CCR6$^+$IgA$^+$B220$^+$ B cells or CCR6$^+$IgA$^+$CCR1$^+$GL7$^-$B220$^+$ B cells in PPs from *Hif1a* cKO and littermate control mice (n=4 per group). **D** Representative histograms and pSmad2/3 expression levels in IgA$^+$B220$^+$ B cells or IgA$^+$CCR1$^+$CCR6$^+$GL7$^-$B220$^+$ SED B cells from *Hif1a* cKO and littermate control mice (n=4 per group). **E** Representative

histograms and αVβ8 expression levels in $CD11b^{+}CD11c^{+}$ dendritic cells from *Hif1a* cKO and littermate control mice (n=4 per group). **F** Representative plots and frequencies of ($CXCR5^{+}PD\text{-}1^{+}Foxp3^{-}CD4^{+}$) Tfh cell and ($CXCR5^{+}PD\text{-}1^{+}Foxp3^{+}CD4^{+}$) Tfr cell in MLN and PPs from *Hif1a* cKO and littermate control mice (n=5 per group). **G** Representative plots and frequencies of ($CD21/35^{+}Podoplanin^{+}CD31^{-}CD45^{-}$) FDC in MLN and PPs from *Hif1a* cKO and littermate control mice (n=5 per group). Data are shown as mean ± SEM. Results are representative of three independent experiments. *p* values were calculated via an unpaired two-tailed Student's *t*-test. NS, not significant ($p > 0.05$).

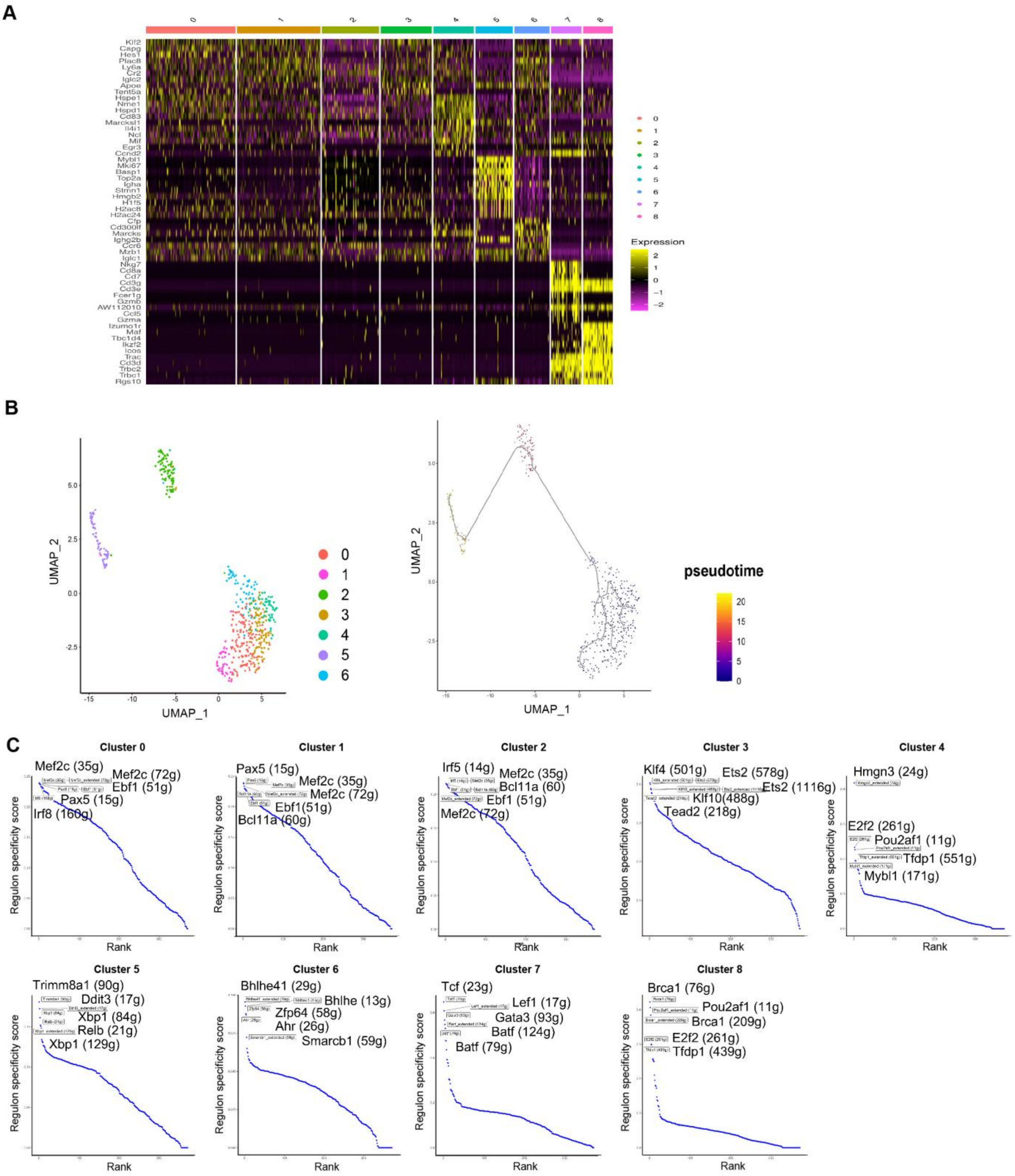

Supplementary figure 4. Pseudotime trajectory and SCENIC analysis in B cell clusters.

A Heatmap of selected differentially expressed marker genes in different B cell subclusters. **B** Display of clustering results for B cell subclusters in UMAP plot and Pseudotemporal ordering (pseudotime) results for trajectory of B cell subclusters by Monocle3. **C** Top5 transcription factor in different cell clusters.

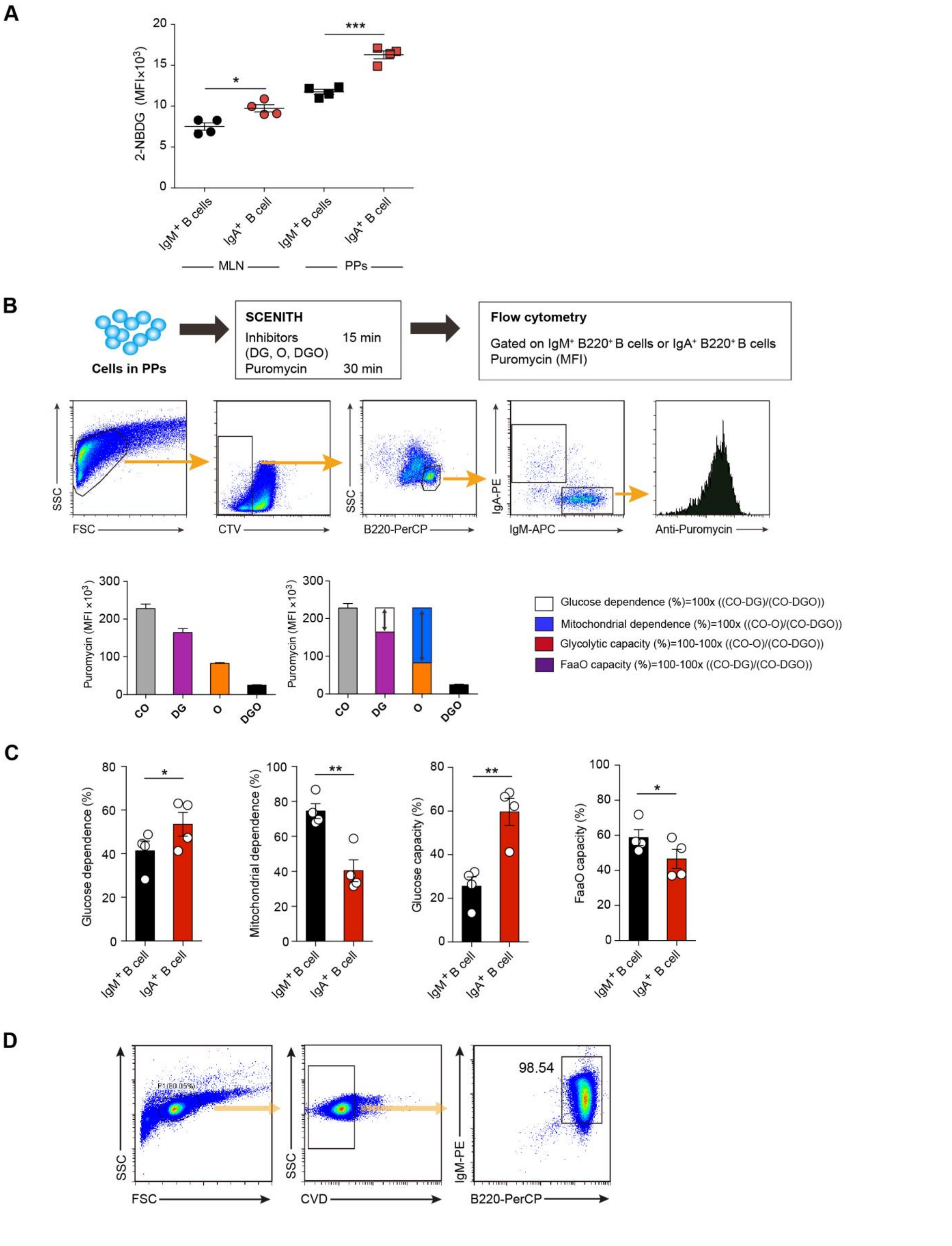

Supplementary figure 5. Metabolic analysis of IgM^{+} B cell and IgA^{+} B cell in MLN and PPs from WT mice.

A 2-NBDG MFI of IgM^+ B cell and IgA^+ B cell in MLN and PPs (n=4). **B**, **C** Schematic description of SCENITH assay on IgM^+ B cell and IgA^+ B cell in PPs (n=4 per group). Puromycin MFI of IgM^+ B cell and IgA^+ B cell in PPs using SCENITH in CO or after the addition of DG, O or DGO. **D** Flow cytometry analysis of surface IgM expression in B cell after B cell enrichment. Data are shown as mean ± SEM. Results are representative of three independent experiments. *p* values were calculated via an unpaired two-tailed Student's *t*-test. $*p < 0.05$, $**p < 0.01$, $***p < 0.001$. NS, not significant ($p > 0.05$).

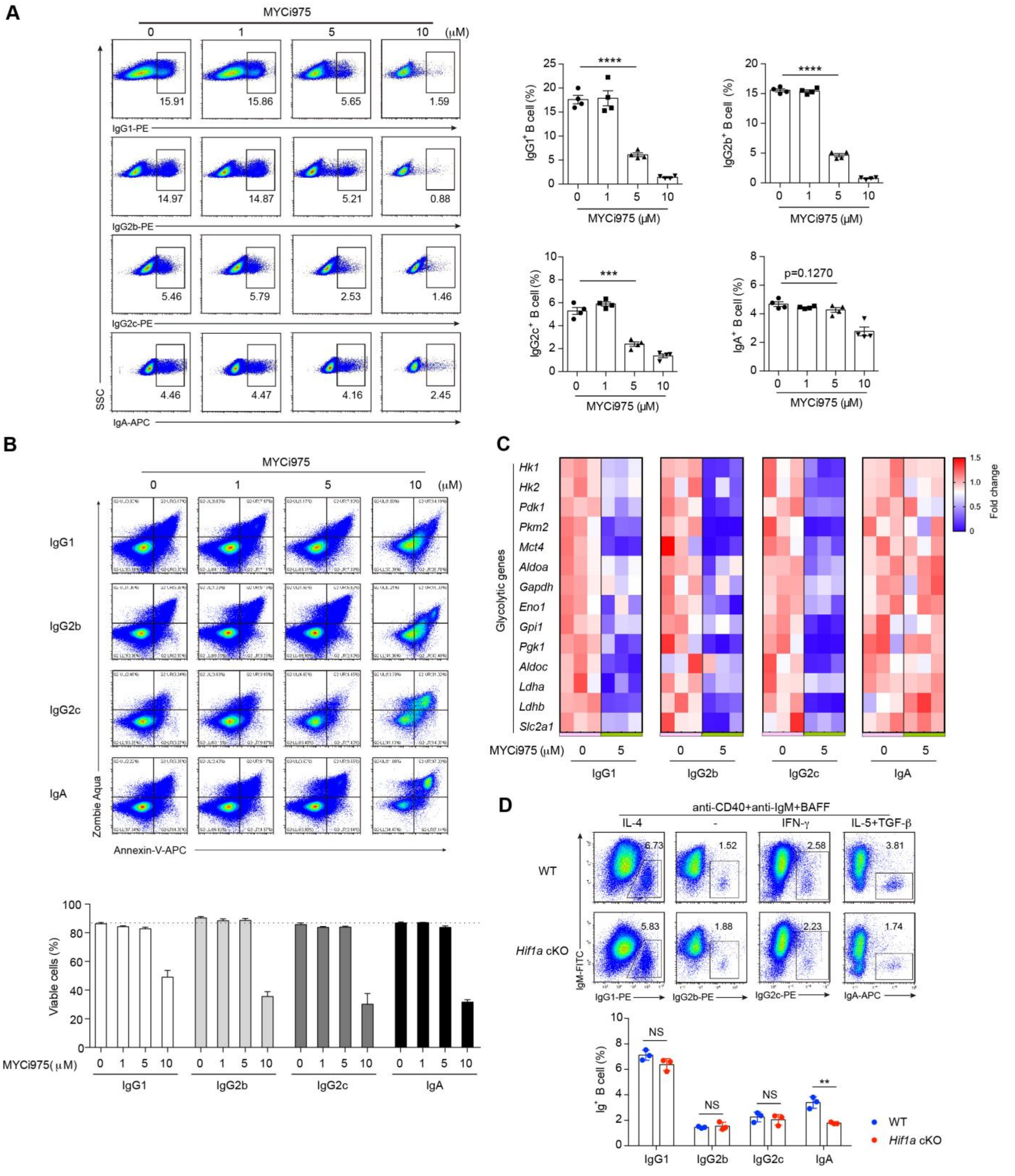

Supplementary figure 6. c-Myc pathway regulates IgG1, IgG2b, IgG2c class switching.

A Representative plots and frequencies of IgG1$^+$, IgG2b$^+$, IgG2c$^+$ or IgA$^+$ B cell in cultured B cell supplemented with vehicle or c-Myc inhibitor MYCi975 (1, 5, 10 μM) under respective class switching condition (n=3 per group). **B** Apoptosis detection by Annexin-V and Zombie Aqua staining in B cell treated with vehicle or MYCi975 (1, 5, 10 μM) after culture in IgG1, IgG2b, IgG2c or IgA class switching conditions (n=3 per group). **C** Heat map displaying glycolytic genes (*Hk1*, *Hk2*,

Pdk1, *Pkm2*, *Mct4*, *Aldoa*, *Gapdh*, *Eno1*, *Gpi1*, *Pgk1*, *Aldoc, Ldha*, *Ladhb, Slc2a1*) expression in vehicle or MYCi975 (5 μM) treated B cells after culture in IgG1, IgG2b, IgG2c or IgA class switching conditions. **D** Flow cytometry analysis of surface IgG1, IgG2b, IgG2c and IgA expression in *Hif1a* KO and WT control B cells after stimulations with anti-CD40 plus anti-IgM for inducing IgG1, IgG2b, IgG2c and IgA class switching respectively (n=3 per group). Data are shown as mean ± SEM. Results are representative of three independent experiments. *p* values were calculated via an unpaired two-tailed Student's *t*-test. $**p < 0.01$, $***p < 0.001$, $****p < 0.0001$. NS, not significant ($p > 0.05$).

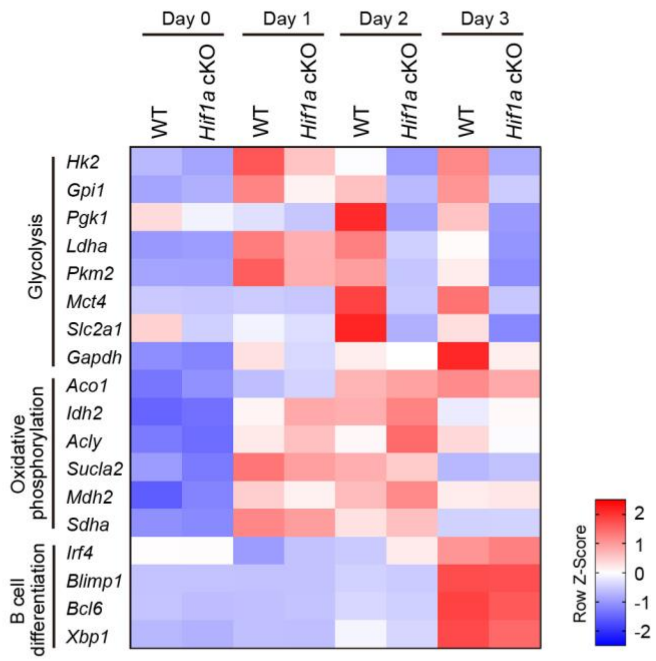

Supplementary figure 7. Gene expression profile of *Hif1a* KO B cells and WT B cells during IgA class switching.

Heat map displaying glycolysis, oxidative phosphorylation and B cell differentiation related genes expression in *Hif1a* KO and WT B cells by qPCR analysis on day 0, day 1, day 2 and day 3 cultured in IgA class switching condition.

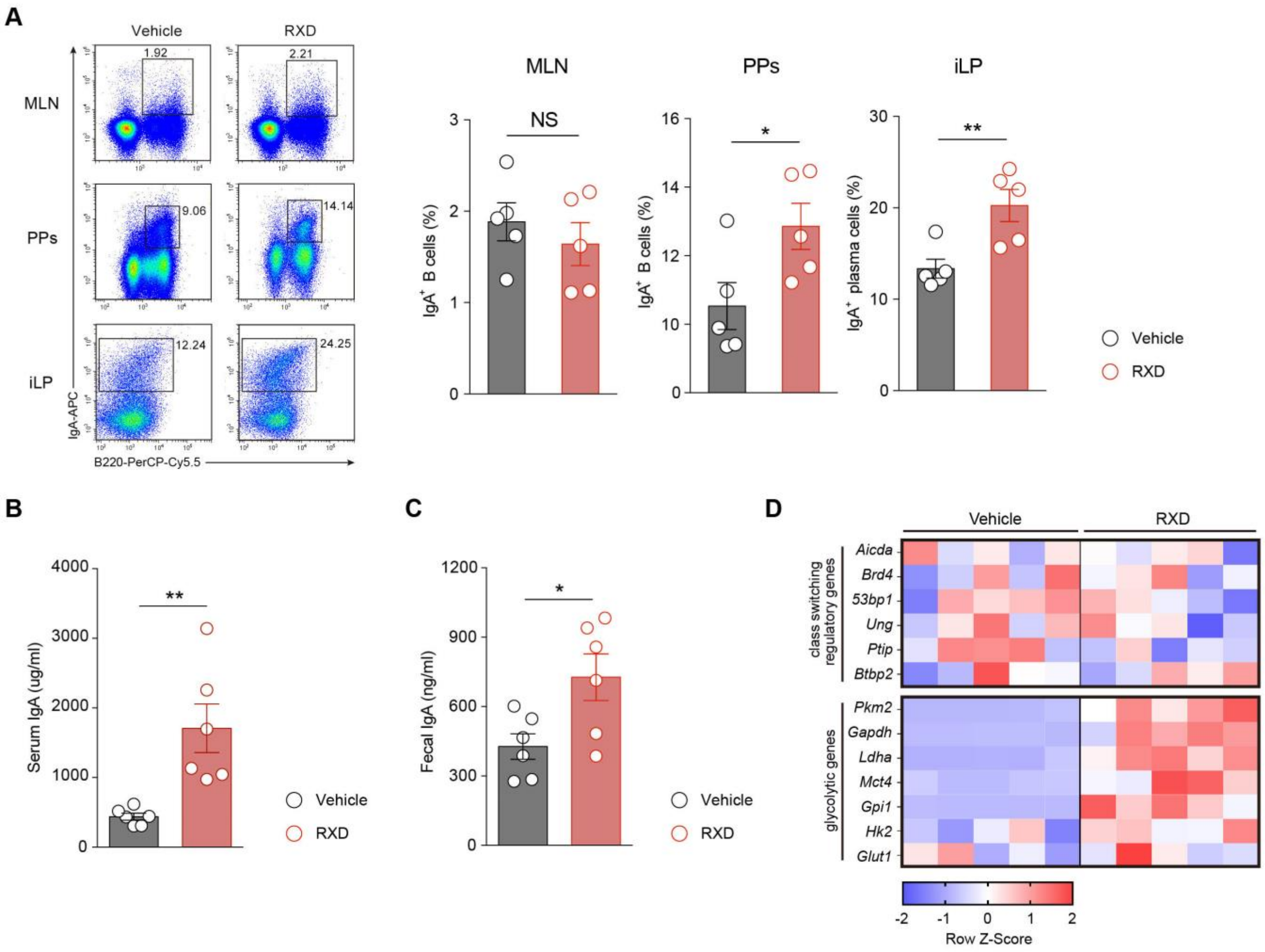

Supplementary figure 8. Effect of RXD treatment on IgA$^+$ B cell differentiation in WT mice.

A Flow cytometry plots and quantification of IgA$^+$ B cells or plasma cells in MLN, PPs and iLP from WT mice after injection with vehicle or RXD (10 mg/kg) for 9 days (n=5 per group). **B** IgA levels in serum from WT mice after injection with vehicle or RXD (10 mg/kg) for 9 days (n=6 per group). **C** IgA levels in cecal contents of mice as shown in (**B**). **D** Heatmap displaying glycolytic and class switching regulatory genes analyzed by qPCR in sorted IgA$^+$ B220$^+$ B cells from WT mice after injection with vehicle or RXD (10 mg/kg) for 9 days (n=5 per group).

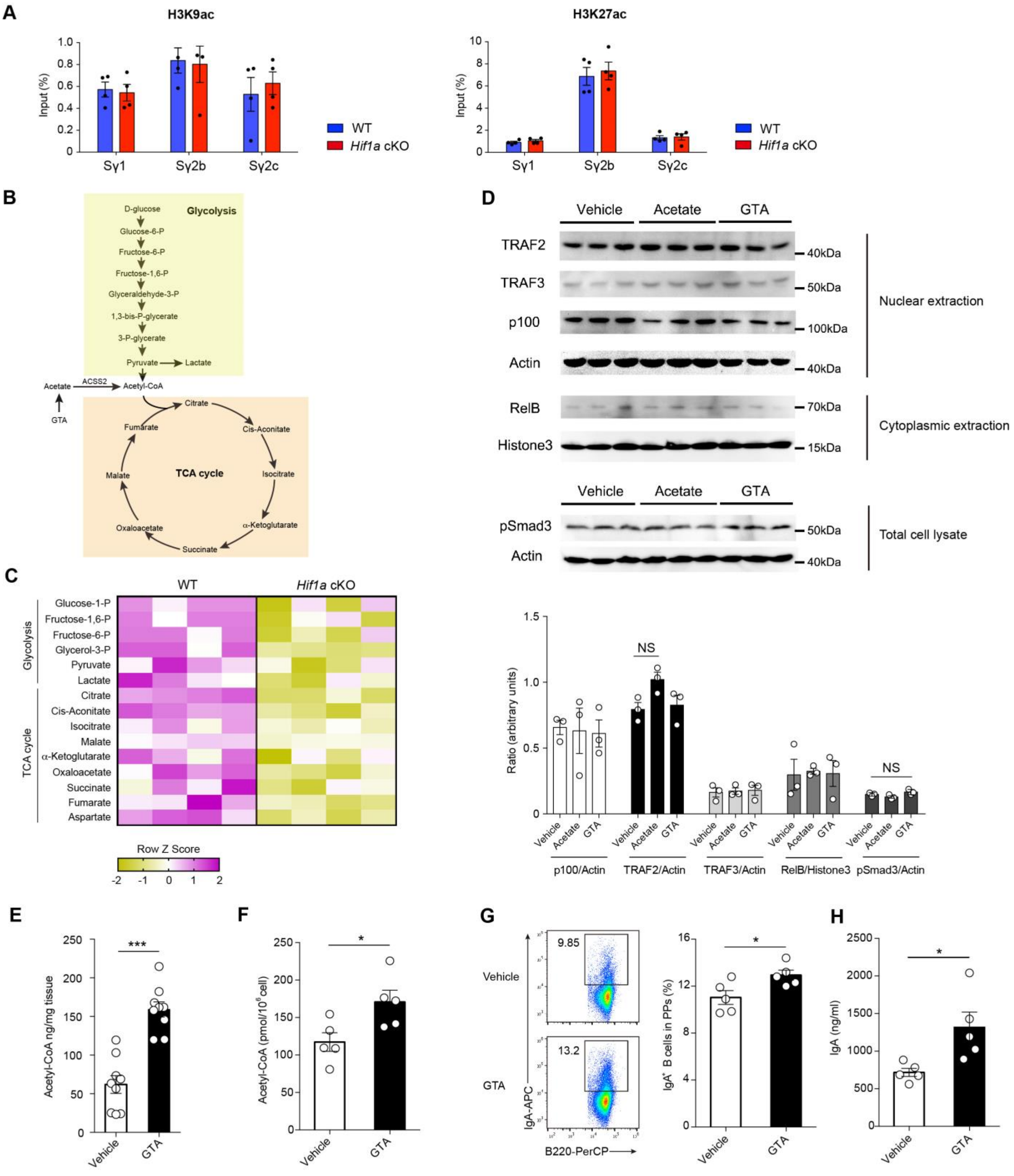

Supplementary figure 9. Epigenetic-metabolic axis in *Hif1a*-deficient B cell and the effect of acetate or GTA treatment on B cell during IgA class switching.

A ChIP-qPCR analysis of H3K9ac and H3K27ac histone modification at Sγ1 region, Sγ2b region and Sγ2c region from *Hif1a* KO and control B cell after culture in IgA class switching condition. **B** Scheme of metabolites in glycolysis and TCA cycles. **C** Heatmap showing relative levels of ^{13}C-labeled metabolites in glycolysis and TCA cycles from *Hif1a* KO and WT B cells under IgA class

switching condition followed by LC-MS analysis (n=4 per group). **D** Immunoblotting analysis of TRAF2, TRAF3, p100, RelB and pSmad3 in cultured B cell during IgA class switching with vehicle, acetate or GTA treatment. Actin and Histone3 were used as the loading controls. Bar graphs represent densitometry results of blotting bands. **E** Acetyl-CoA levels in colonic tissue from WT mice treated for 9 days with vehicle or GTA (n=9 per group). **F** Acetyl-CoA levels in pooled B cells from MLN and PPs of WT mice treated for 9 days with vehicle or GTA (n=5 per group). **G** Flow cytometry analysis and quantification of $B220^{+}IgA^{+}$ B cells in PPs from C57BL/6 WT mice treated for 9 days with vehicle or GTA (n=5 per group). **H** ELISA analysis of IgA levels in cecal contents of C57BL/6 WT mice treated for 9 days with vehicle or GTA. Data are shown as mean ± SEM. Results are representative of three independent experiments. *p* values were calculated via an unpaired two-tailed Student's *t*-test (**D**-**H**). $*p < 0.05$, $***p < 0.001$. NS, not significant ($p > 0.05$).

Supplementary Table 1. List of antibodies used for flow cytometry.

Protein name	Flurochrome	Company	Clone	Cat #	RRID
CD4	BV421	BD	GK1.5	562891	AB_2737870
CD8	APC-CY7	BD	53-6.7	557654	AB_396769
CD11b	BV605	Biolegend	M1/70	101237	AB_11126744
CD95	PE-CY7	BD	Jo2	552811	AB_396768
GL7	AF647	Biolegend	Biolegend	GL7	AB_2562185
B220	PerCP-Cy5.5	BD	RA3-6B2	552771	AB_394457
B220	PerCP	BD	RA3-6B2	553093	AB_394622
CD19	APC	ThermoFisher	1D3	17-0193-80	AB_1659678
IgA	APC	SouthernBiotech	11-44-2	1165-11	AB_ 2794661
IgG1	PE	BD	A85-1	550083	AB_393553
IgG2b	PE	Biolegend	RMG2b-1	406707	AB_2563380
IgG2c	Biotin	BD	5.7	553504	AB_394889
IgM	FITC	ThermoFisher	II/41	11-5790-81	AB_465244
IgD	APC/Cy7	Biolegend	11-26c.2a	405716	AB_10662544
F4/80	PE	Biolegend	BM8	123110	AB_893486
Gr1	PerCP	Biolegend	RB6-8C5	108426	AB_893557
CD185	APC/Cy7	Biolegend	L138D7	145526	AB_2566799
CD138	APC	BD	281-2	561705	AB_1645216
CD45	AF700	Biolegend	30-F11	103128	AB_493715
CD31	PE	Biolegend	W18222B	160203	AB_493715
CD21/35	FITC	BD	7G6	553818	AB_395070
Podoplanin	APC	Biolegend	8.1.1	127409	AB_10612940
CD279	PE	Biolegend	RMP1-30	109104	AB_313421
Foxp3	APC	ThermoFisher	FJK-16s	17-5773-82	AB_469457
B220	FITC	BD	RA3-6B2	553088	AB_394618
CD4	BV421	Biolegend	RM4-5	100563	AB_2563052
CD196	PE	Miltenyibiotec	REA277	130-126-497	AB_2655945
CD11b	APC	Biolegend	M1/70	101212	AB_312795
CD23	PE	BD	B3B4	553139	AB_394654
CD16/32		Biolegend	93	101302	AB_312801
Streptavidin	PE	BD		554061	AB_10053328
Puromycin	FITC	Merck	12D10	MABE343	
HIF-1a	PE	R&D	241812	IC1935P	
Annexin-V	APC	Biolegend		640941	AB_2616657

Supplementary Table 2. List of primers used for qPCR and ChIP-qPCR primers.

Primer pair name	Sequence (5'-3')	Application
Hk2 (For)<br>*Hk2* (Rev)	CCCTGTGAAGATGTTGCCCACT<br>CCTTCGCTTGCCATTACGCACG	qPCR
Gpi1 (For)<br>*Gpi1* (Rev)	GTTGCCTGAAGAGGCCAGG<br>GCTGTTGCTTGATGAAGCTGATC	qPCR
Pdk1 (For)<br>*Pdk1* (Rev)	GATTCAGGTTCACGTCACGCT<br>GACGGATTCTGTCGACAGAG	qPCR
Ldha (For)<br>*Ldha* (Rev)	CACAAGCAGGTGGTGGACAG<br>AACTGCAGCTCCTTCTGGATTC	qPCR
Pkm2 (For)<br>*Pkm2* (Rev)	CAGGAGTGCTCACCAAGTGG<br>CATCAAGGTACAGGCACTACAC	qPCR
Mct4 (For)<br>*Mct4* (Rev)	TGGCATCTCATATGGCATGGTG<br>CACCTCCTCAGGCTCTGTC	qPCR
Slc2a1 (For)<br>*Slc2a1* (Rev)	TTAATCGCTTTGGCAGGCGG<br>CCCAGTTTGGAGAAGCCCAT	qPCR
Ldhb (For)<br>*Ldhb* (Rev)	CCTCAGATCGTCAAGTACAGCC<br>ATCCGCTTCCAATCACACGGTG	qPCR
Pgk1 (For)<br>*Pgk1* (Rev)	GATGCTTTCCGAGCCTCACTGT<br>ACCAGCCTTCTGTGGCAGATTC	qPCR
Aldoc (For)<br>*Aldoc* (Rev)	GGCAGAGATGAACGGGCTTG<br>GGCGATGTAGAGGGACTGTG	qPCR
Eno1 (For)<br>*Eno1* (Rev)	TGCGTCCACTGGCATCTAC<br>CAGAGCAGGCGCAATAGTTTTA	qPCR

Aldoa (For)<br>*Aldoa* (Rev)	CACGAGACACTGTACCAGAAGG<br>TTGTCTCGCCATTGGTTCCTGC	qPCR
Hk1 (For)<br>*Hk1* (Rev)	GAAAGGAGACCAACAGCAGAGC<br>TTCGTTCCTCCGAGATCCAAGG	qPCR
Gapdh (For)<br>*Gapdh* (Rev)	CATCACTGCCACCCAGAAGACTG<br>ATGCCAGTGAGCTTCCCGTTCAG	qPCR
G6pdx (For)<br>*G6pdx* (Rev)	GACCAAGAAGCCTGGCATGTTC<br>AGACATCCAGGATGAGGCGTTC	qPCR
Pgd (For)<br>*Pgd* (Rev)	CATCGCTGCAAAAGTGGGAACC<br>AGCCTCACAGATGAGCTGCATG	qPCR
Tkt (For)<br>*Tkt* (Rev)	GCTAACATCCGAATGCCTACGC<br>TTGGTGTCTCCATCCAGGGCAA	qPCR
Sdha (For)<br>*Sdha* (Rev)	GGGAAGATTACAAAGTGCGGG<br>TTCCCCAAACGGCTTCTTCT	qPCR
Sdhb (For)<br>*Sdhb* (Rev)	GTGGATCTGAATAAGTGCGGA<br>CCAGAGTATTGCCTCCGTTGA	qPCR
Sdhc (For)<br>*Sdhc* (Rev)	CGACACTTGCTATGGGACCTA<br>AACACAGCAAGAACCACGAC	qPCR
Idh2 (For)<br>*Idh2* (Rev)	GACCGCTATCTCTCCATCACCT<br>GTTGGAAGCAGATGTGACCGTC	qPCR
Mdh2 (For)<br>*Mdh2* (Rev)	TCACTCCTGCTGAAGAACAGCC<br>CCTTTGAGGCAATCTGGCAACTG	qPCR
Idh1 (For)<br>*Idh1* (Rev)	CAGGCTCATAGATGACATGGTGG<br>CACTGGTCATCATGCCAAGGGA	qPCR
Aco1 (For)<br>*Aco1* (Rev)	CCATCCGTGATGTTAGGAGCAG<br>GACAGGTAAGGCATGACTCCAC	qPCR
Acly (For)	AGGAAGTGCCACCTCCAACAGT	qPCR

Acly (Rev)	CGCTCATCACAGATGCTGGTCA	
Sucla2 (For)<br>*Sucla2* (Rev)	GGTGTCTCTGTTCCCAAAGGCT<br>TTTCCTCTGCCGCCAGCCAAAA	qPCR
mGLT (For)<br>*mGLT* (Rev)	CTCTGGCCCTGCTTATTGTTG<br>AATGGTGCTGGGCAGGAAGT	qPCR
g1GLT (For)<br>*g1GLT* (Rev)	GGCCCTTCCAGATCTTTGAG<br>GGATCCAGAGTTCCAGGTCACT	qPCR
g2bGLT (For)<br>*g2bGLT* (Rev)	CACTGGGCCTTTCCAGAACTA<br>CACTGAGCTGCTCATAGTGTA	qPCR
g2cGLT (For)<br>*g2cGLT* (Rev)	GAAGGTTCATCGGGAAAGGC<br>GCCAGTTGTATCTCCACACACAG	qPCR
eGLT (For)<br>*eGLT* (Rev)	ACTAGAGATTCACAACGCCTGGGA<br>AGGGTCATGGAAGCAGTGCCTTTA	qPCR
aGLT (For)<br>*aGLT* (Rev)	GCCATCAAGGCAGGGCCTGGG<br>GCAGGCAGGGCTAGATATGG	qPCR
14-3-3 (For)<br>*14-3-3* (Rev)	GGCCGCGGCCATGAAGAACG<br>GGAGCGGCGAGCCCCCACCACG	qPCR
Mbip (For)<br>*Mbip* (Rev)	GGTCACAAACCTACAGGCATGC<br>GGTAGATGTCTCTTGGTACTGGG	qPCR
Ada3 (For)<br>*Ada3* (Rev)	GCTTGAGACTCTGCTGTCCTCT<br>CGACCTAGCTTCAGGAATCGTC	qPCR
Spt3 (For)<br>*Spt3* (Rev)	AAGGCATTGACGAGGATGACCTC<br>AGCTCTCCTGTCTGGTCGATAG	qPCR
Rbbp5 (For)<br>*Rbbp5* (Rev)	CAGAGCCTATGCAGAAGCTGCA<br>CTTCACCAGGTTGCCAATGCTC	qPCR
Cxxc1 (For)<br>*Cxxc1* (Rev)	GGATGAGCGATGCAGAAGAGTC<br>CGATGCCGTTTGTACCTCTCCT	qPCR

Wdx5 (For)<br>*Wdx5* (Rev)	CTCCTTGTGTCTGCCTCTGATG<br>CCTGAGACGATGAGGTTGGACT	qPCR
Mll1 (For)<br>*Mll1* (Rev)	CACCATTGGCAATGAGCGGTTC<br>AGGTCTTTGCGGATGTCCACGT	qPCR
Paxip (For)<br>*Paxip* (Rev)	CACCATTGGCAATGAGCGGTTC<br>AGGTCTTTGCGGATGTCCACGT	qPCR
Mll2 (For)<br>*Mll2* (Rev)	GGAAGCCAGATGAAAGGACTCC<br>TGGTCCAAGGATGGAGGCAACA	qPCR
Msh2 (For)<br>*Msh2* (Rev)	GAACAAAGGCGAGTATGAAGAGG<br>GCGTCTAAGTGAGCCAGCACAT	qPCR
Ptip (For)<br>*Ptip* (Rev)	CCCACTTTCACAAGCCGATGTAC<br>CTTCAGCACTGTGTTGAGCCAG	qPCR
Aicda (For)<br>*Aicda* (Rev)	TCTGCTACGTGGTGAAGAGGAG<br>CCAGTCTGAGATGTAGCGTAGG	qPCR
53bp1 (For)<br>*53bp1* (Rev)	CTGGTTAGTCCTGAGACAGAGG<br>AGATGCAGCCAAACACAGGCAC	qPCR
Ung (For)<br>*Ung* (Rev)	ATGGACCCAGATGTGCGACATC<br>CTGGTCTTTGGACACTGAAGCAG	qPCR
Il1b (For)<br>*Il1b* (Rev)	CAGGCAGGCAGTATCACTCA<br>AGGTGCTCATGTCCTCATCC	qPCR
Il6 (For)<br>*Il6* (Rev)	TCCTTCCTACCCCAATTTCC<br>GCCACTCCTTCTGTGACTCC	qPCR
Tnf (For)<br>*Tnf* (Rev)	AAACACAAGATGCTGGGACA<br>TTGATGGTGGTGCATGAGAG	qPCR
Cxcl1 (For)<br>*Cxcl1* (Rev)	TCCAGAGCTTGAAGGTGTTGCC<br>AACCAAGGGAGCTTCAGGGTCA	qPCR
Ccl2 (For)	GCTACAAGAGGATCACCAGCAG	qPCR

Ccl2 (Rev)	GTCTGGACCCATTCCTTCTTGG	
Ccl3 (For)<br>*Ccl3* (Rev)	ACTGCCTGCTGCTTCTCCTACA<br>ATGACACCTGGCTGGGAGCAAA	qPCR
Il10 (For)<br>*Il10* (Rev)	AGGGGAGAAATCGATGACAG<br>CCAAGCCTTATCGGAAATGA	qPCR
Sγ1 (For)<br>Sγ1 (Rev)	CAGGTGCTGCAGCTACATACGGG<br>AGTCCCACAATTCTACCTCTCCT	ChIP-qPCR
Sγ2b (For)<br>Sγ2b (Rev)	AGCTCCAAAAGCTCAGCAGAC<br>AGCCCCAGCTTACAAAGAGCT	ChIP-qPCR
Sγ2c (For)<br>Sγ2c (Rev)	TTCGGGACCCACAGTACATTTGTGATG<br>AGAGCTGTAATGCCTGGTTGCCTCCTA	ChIP-qPCR
Sα (For)<br>Sα (Rev)	GATTCAGGGAGCAAGAGCCA<br>CCTCTGTCTAGCCTGGGAGT	ChIP-qPCR
